# The Glycolytic Gatekeeper PDK1 defines different metabolic states between genetically distinct subtypes of human acute myeloid leukemia

**DOI:** 10.1038/s41467-022-28737-3

**Published:** 2022-03-01

**Authors:** Ayşegül Erdem, Silvia Marin, Diego A. Pereira-Martins, Roldán Cortés, Alan Cunningham, Maurien G. Pruis, Bauke de Boer, Fiona A. J. van den Heuvel, Marjan Geugien, Albertus T. J. Wierenga, Annet Z. Brouwers-Vos, Eduardo M. Rego, Gerwin Huls, Marta Cascante, Jan Jacob Schuringa

**Affiliations:** 1 Department of Experimental Hematology, University Medical Center Groningen, University of Groningen, Hanzeplein 1, 9700 RB Groningen, The Netherlands; 2 Department of Biochemistry and Molecular Biology, Faculty of Biology, Avinguda Diagonal 643, 08028 Barcelona, Spain; 3 CIBER of Hepatic and Digestive Diseases (CIBEREHD), Institute of Health Carlos III, 28029 Madrid, Spain; 4 Institute of Biomedicine of University of Barcelona, 08028 Barcelona, Spain; 5 Hematology Division, LIM31, Faculdade de Medicina, University of São Paulo, São Paulo, SP Brazil; 6 Department of Laboratory Medicine, University Medical Center Groningen, University of Groningen, Hanzeplein 1, 9700 RB Groningen, The Netherlands

**Keywords:** Cancer metabolism, Acute myeloid leukaemia

## Abstract

Acute myeloid leukemia remains difficult to treat due to strong genetic heterogeneity between and within individual patients. Here, we show that Pyruvate dehydrogenase kinase 1 (PDK1) acts as a targetable determinant of different metabolic states in acute myeloid leukemia (AML). PDK1^low^ AMLs are OXPHOS-driven, are enriched for leukemic granulocyte-monocyte progenitor (L-GMP) signatures, and are associated with *FLT3*-ITD and *NPM1*cyt mutations. PDK1^high^ AMLs however are OXPHOS^low^, wild type for *FLT3* and *NPM1*, and are enriched for stemness signatures. Metabolic states can even differ between genetically distinct subclones within individual patients. Loss of PDK1 activity releases glycolytic cells into an OXPHOS state associated with increased ROS levels resulting in enhanced apoptosis in leukemic but not in healthy stem/progenitor cells. This coincides with an enhanced dependency on glutamine uptake and reduced proliferation in vitro and in vivo in humanized xenograft mouse models. We show that human leukemias display distinct metabolic states and adaptation mechanisms that can serve as targets for treatment.

## Abstract (web summary)

Acute myeloid leukemia (AML) is genetically a very heterogeneous disease. Here, Erdem et al. uncover heterogeneity in the metabolic landscape of AML and identify Pyruvate dehydrogenase kinase 1 (PDK1) as a targetable determinant of different metabolic states in distinct subtypes of AML.

## Introduction

Acute myeloid leukemia (AML) is a heterogeneous disease, arising as a consequence of a series of genetic abnormalities in the hematopoietic stem/progenitor compartment^1–3^. The repertoire of initiating founder mutations and secondary driver mutations in AML is rather patient-specific and can also result in complex clonal heterogeneity within individual patients^4–10^. As a consequence, the cell biological characteristics differ significantly between AML patients, and even between genetically distinct subclones within patients. Therefore, to improve therapy outcome for this dismal disease, a personalized treatment approach will be required.

It has long been noted that cancer cells differ in their metabolome from healthy cells. Warburg proposed that cancer cells would rely on glycolysis as the primary mode for energy production, which is quicker than oxidative phosphorylation (OXPHOS) in ATP generation and provides for the anabolic needs of proliferating cells^11^. Both normal hematopoietic stem cells (HSCs) and leukemic stem cells (LSCs) are thought to reside in relatively hypoxic bone marrow niches, conditions that might favor a glycolytic over an OXPHOS state. Indeed, it has been shown that dormant HSCs utilize glycolysis instead of mitochondrial oxidative phosphorylation^12,13^, in part via a MEIS1-controlled expression of HIF1^14^. Lowering mitochondrial activity of HSCs has been shown to enhance self-renewal^15–18^. Even though HSCs appear to contain a considerable number of mitochondria, they are relatively inactive^19,20^. HSCs maintain low levels of reactive oxygen species (ROS) in order to maximize their lifespan^21^, and also leukemic stem cells have been proposed to be glycolytic and maintain a ROS^low^ state^13,22^, although this might also depend on mutational status^23^. Recent work also indicated that leukemic stem cells might in fact be sensitive to OXPHOS inhibition^24–27^, an observation mimicked in healthy HSCs where complete removal of respiratory chain activity resulted in reduced HSC function^28^.

Pyruvate dehydrogenase kinases (Pdks) have been shown to act as gatekeepers of glycolysis in murine long-term HSCs (LT-HSCs) thereby maintaining them in a quiescent state^29^. Pdks phosphorylate Pyruvate Dehydrogenase (PDH) thereby inactivating the conversion of pyruvate into acetyl-CoA. Consequently, pyruvate will be anaerobically converted into lactate. Pdks are highest expressed in the most immature stem/progenitor cell compartment^29^. At the protein level it appeared that in particular PDK1 and PDK3 were higher expressed in the LSK (Lineage^−^Sca1^+^c-kit^+^) cells compared to more differentiated cells^30^. Indeed, silencing Pdk1 impaired engraftment of primitive HSCs^31^.

Evidence is accumulating that changes in the regulation of energy fluxes is an important hallmark of malignant transformation of HSCs to LSCs. Although molecular events dictating leukemia-specific alterations in metabolic programs are far from clear, several metabolic signatures have recently been identified to directly influence leukemic blast survival in acute myeloid leukemia (AML). The glucose metabolism metabolites lactate and pyruvate are elevated in AML patient serum samples, and are strong predictors of poor prognosis^32^. Yet, given the rather heterogeneous genetic landscape of AML, it appears likely that differences exist in metabolic reprogramming between individual patients. Here, we show that metabolically distinct subtypes of AML can be identified by high expression of PDK1, associated with low OXPHOS and an increase in stemness transcriptional signatures. Insight into such differences will further aid our molecular and cell biological understanding of clonal heterogeneity in AML and will also provide means for personalized and subclone-specific targeting strategies.

## Results

### Profiling of central energy metabolism reveals heterogeneity in metabolic signatures in AML compared to healthy HSPCs

**Fig. 1:**
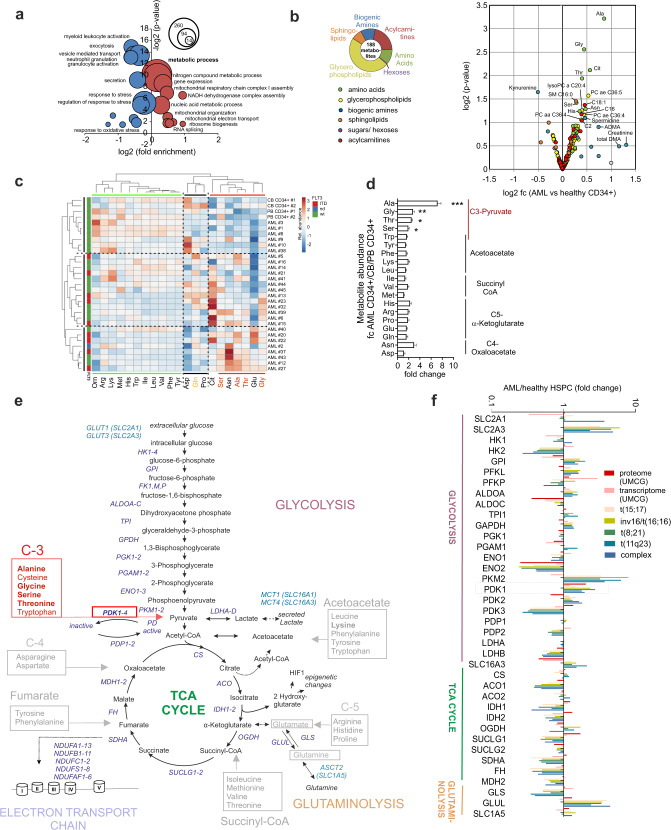
Integrative metabolome, proteome and transcriptome profiling of main energy fluxes in AML and normal hematopoietic stem and progenitors. **a** Bubble chart displaying gene ontology (GO) enrichment analysis of the label-free quantified proteins in AML CD34^+^ (*n* = 42) fraction versus healthy PB CD34^+^ cells (*n* = 6). Bubble sizes represent numbers of proteins contributing to each cellular pathway, upregulated pathways in AML are in red and downregulated pathways are labeled in blue; *x*-axis and *y*-axis indicate log2 fold-changes in up/downregulated GO terms in AML and the FDR *p*-value, respectively. **b** Pie chart showing six metabolite families screened using LC-MS/MS targeted approach in the AML CD34^+^ (*n* = 27) fraction versus healthy PB CD34^+^ cells (*n* = 2) and CB CD34^+^ cells (*n* = 2). The volcano plot compares 188 metabolites in AML CD34^+^ versus healthy CB and PB CD34^+^. Each point represents a single metabolite and is colored by corresponding metabolite family. **c** Heatmap of amino-acid abundance (*x*-axis) and their associations with CD34^+^ AML primary individuals and healthy PB (peripheral blood stem cells, PBSC) and CB (cord blood)-derived CD34^+^ samples. *y*-axis indicates genetic backgrounds of AML patients with either FLT3-ITD mutated or wild type. Color code for higher abundance is red and for lower abundance is blue (each value was normalized to the average of each metabolite in each AML or healthy group). Dotted lines separates major heterogeneous differences in amino-acid metabolism. **d** Bar plot showing the fold-changes in the internal abundance of each amino acids in AML CD34^+^ compared to healthy PB/CB (peripheral blood mobilized stem cells and cord blood)-derived CD34^+^. (*p*-values; 0.00061 for Ala, 0.00277 for Gly, 0.038 for Ser, 0.01 for Thr). Error bars represent mean ± SEM. **e** Schematic representation of the main energy flux of cells starting with glycolysis and continues with TCA cycle and glutaminolysis. Enzymes are indicated in purple, membrane transporters are indicated in blue, amino acids involved in the main metabolism indicated in gray and/or red. **f** Integration of our proteome, UMCG transcriptome and MILE transcriptome data for the enzymes involved in Glycolysis, TCA cycle and Glutaminolysis. Fold-changes were colored according to log2 transformed expression values after compared to normal HSPCs. *p < 0.05; **p < 0.01; ***p < 0.001, **b**, **d** Student’s *t*-test (two-sided).

To better understand derailed signaling in AML we performed extensive quantitative proteome and transcriptome analyses on a large cohort of primary AML patient samples and healthy CD34^+^ control hematopoietic stem/progenitor cells (HSPCs) and compared that with high-throughput LC-MC/MS (liquid chromatography-tandem mass spectrometry)-based targeted metabolomics analysis. Our full label-free quantitative proteome analyses within the CD34^+^ fraction (or mononuclear blasts in case of *NPM1* mutant AML)^7^ in primary AML patient samples (*n* = 42, Supplementary Data 1) compared to healthy CD34^+^ hematopoietic stem/progenitor cells revealed that downregulated proteins in AML were significantly enriched for gene ontology (GO) terms associated with myeloid commitment, while upregulated proteins were enriched for GO terms “RNA splicing”, “ribosome biogenesis” and various metabolic processes (Fig. 1a and Supplementary Data 2). Among the upregulated proteins we identified several that are part of the electron chain transport, potentially linked to increased mitochondrial respiration, but we also identified PDK1 to be among the upregulated proteins (Supplementary Data 2), which would potentially lead to a more glycolytic metabolism as PDK1 has been shown to act as the gatekeeper of glycolysis by phosphorylating and thereby inactivating Pyruvate Dehydrogenase (PDH), subsequently preventing the conversion of pyruvate into acetyl-CoA and TCA cycle entry. These potentially opposing phenotypes suggested that clear differences in metabolic profiles across different subtypes of AML might exist, which we wished to explore further.

First, we performed a metabolome screen in primary AML patient samples using the AbsoluteIDQ p180 Biocrates kit in which up to 188 endogenous metabolites can be quantified across six metabolite classes (hexoses, amino acids, biogenic amines, acylcarnitines, glycerophospholipids, sphingolipids) (Fig. 1b). AML CD34^+^ blasts (*n* = 27) were compared to healthy cord blood (CB) and mobilized PB (PBSC) CD34^+^ cells (*n* = 4). The most striking differences were observed in amino-acid (AA) metabolism. A significantly higher abundance of alanine, glycine, threonine and serine amino-acids was observed (Fig. 1b–d). These four AAs are classified as C-3 family AAs and act as precursors of pyruvate, a key intersection metabolite of glycolysis towards either the Krebs cycle (also known as the citric acid cycle or tricarboxylic acid (TCA) cycle) or lactate fermentation (Fig. 1e). This group of AAs clustered away from other subgroups (the acetoacetate, succinyl CoA, C5-α-ketoglutarate and C4-oxaloacetate subgroups) that are more associated with the TCA cycle (Fig. 1c, d). Furthermore, an inverse correlation was noted between pyruvate-related C3 AAs and—among others—the level of branched chain AAs leucine, isoleucine and valine (Fig. 1c). Ornithine, citrulline and arginine are linked via the urea cycle and while ornithine and arginine clustered closely together, citrulline did not. Intracellular glutamine levels were among the most variable between individual AML patients, suggesting that the level of glutaminolysis differs considerably (Fig. 1c). While no associations were found with *IDH1*/*2* mutational status, we observed that, in line with previous studies^33^, glutamine levels were higher in FLT3-ITD patients compared to FLT3 wild type AMLs (Supplementary Fig. 1).

The acylcarnitine levels of C16 and C18:1 were significantly increased in AML CD34^+^ cells, largely driven by the presence of FLT3-ITDs, suggesting differences in free fatty acid oxidation in these types of AML (Supplementary Fig. 2a). The levels of biogenic amines, glycerophospolipids (which includes phosphatidylcholines (PC) and lysophosphatidylcholines (lysoPC)) and sphingolipids were rather comparable between AML CD34^+^ and healthy CD34^+^ cells (Supplementary Fig. 2b–d).

To be able to interpret metabolic differences between AML and healthy CD34^+^ cells we compared our quantitative proteome data with transcriptome data generated by us^34^ and others^3,35^, specifically focusing on glycolysis, the TCA cycle and glutaminolysis (Fig. 1e, f). Overall, TCA-related enzymes were often downregulated in AML versus healthy CD34^+^ cells, while various glycolysis-related enzymes were upregulated. In particular Pyruvate Dehydrogenase Kinase 1 (PDK1) expression was significantly and consistently upregulated in AMLs across independent datasets (Fig. 1f), in line with our initial proteome studies. Furthermore, mouse models have shown that Pdks control LT-HSC quiescence and metabolism linked to higher pyruvate metabolite abundance^29^. Since our metabolome screen identified the strongest differences in C-3 AAs, which can act as precursors for pyruvate, we wished to study the role of PDKs in AML in further detail as these are described to control the fate of this metabolite.

### PDK1 is highly expressed in AML and modulates oxidative respiration activity

**Fig. 2:**
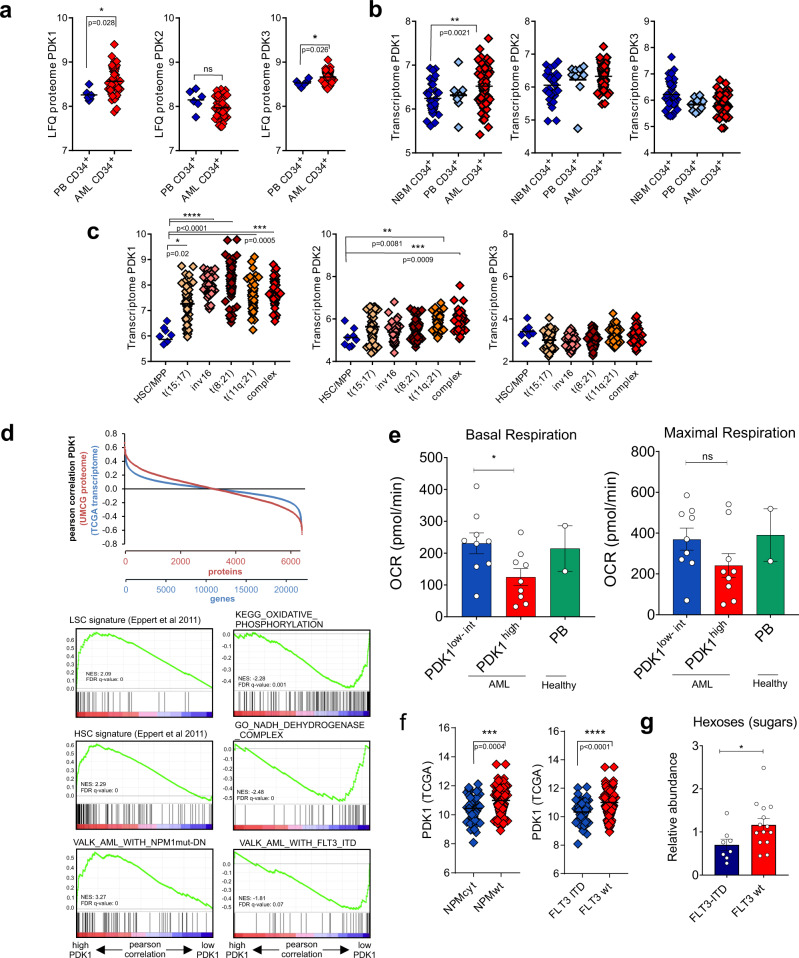
PDK1 is upregulated in AML and is associated with reduced oxidative phosphorylation. **a** PDK1, PDK2, PDK3 (log10) protein expression in healthy primary PB CD34^+^ cells (*n* = 6) and primary AML CD34^+^ cell fraction (*n* = 42). Each square represents one sample, and data are presented as mean by horizontal lines. **b** mRNA expression (log 2) of *PDK1, PDK2, PDK3* in normal bone marrow (NBM) (*n* = 33), PB (*n* = 9) and AML CD34^+^ cells (*n* = 66), and data are presented as mean by horizontal lines. **c** Transcriptome data (log 2) retrieved from the Bloodspot site (GSE13159), comparison for *PDK1, PDK2* and *PDK3* in healthy HSC/MPP compared to AML with t(15;17), inv 16, t(8;21), t(11q;21) and complex phenotype (*n* = 252), and data are presented as mean by horizontal lines. **d** Pearson correlation of PDK1 expression versus quantitative proteome (*n* = 42) and TCGA transcriptome dataset (*n* = 173). GSEA plots for enriched molecular signatures in PDK1^high^ versus PDK1^low^ proteome/transcriptome sets are also shown. Normalized enrichment scores (NES) and false discovery rate (FDR) *q*-values (FDR q) are included. **e** Oxygen consumption rate (OCR) measured with Seahorse assay under basal and maximal conditions. Bar graphs show OCR levels in healthy PB CD34^+^ cells (*n* = 2 independent samples, each dot shows mean of quadruplicate technical replicates), PDK1^low/int^ AML CD34^+^ primary patient cells (*n* = 9 independent samples,each dot shows mean of quadruplicate technical replicates) compared to PDK1^high^ AML CD34^+^ primary patient cells (*n* = 9, measured in quadruplicates) based on protein expression (*p* = 0.040, ns = not significant). **f** TCGA mRNA expression of *PDK1* in NPM1-mutated and FLT3-ITD mutated and wild type AMLs (left panel and right panel, respectively) (*n* = 173). Data are presented as mean. **g** Hexoses metabolite family internal abundance in FLT3-ITD mutated AML primary CD34^+^ samples (*n* = 8) compared to FLT3-wt CD34^+^ AML samples (*n* = 14) (*p* = 0.035). Error bars represent mean ± SEM. *p < 0.05, **p < 0.01, ***p < 0.001, **a**, **f**, **g** Student’s *t*-test (two-sided) or **b**, **c**, **e** Kruskal–Wallis one-way ANOVA test for multiple comparisons.

The PDK family consists of 4 members, PDK1-4. PDK4 remained below detection levels, while PDK1 was most consistently upregulated across multiple independent proteome (Fig. 2a) and transcriptome (Fig. 2b, c) datasets, as well as across multiple distinct genetic subgroups of AML (Fig. 2c). Yet, a strong variation in *PDK1* expression was noted as well, suggesting significant heterogeneity between individual AML patients. To address this further we performed Pearson correlation studies using our quantitative proteome (*n* = 42) and the TCGA transcriptome dataset (*n* = 173)^3^, ranked expression correlated to *PDK1*, and performed gene set enrichment analyses (GSEA) (Fig. 2d). These studies revealed that AML samples with high PDK1 expression were significantly enriched for leukemic stem cell and hematopoietic stem cell signatures, while AMLs with the lowest PDK1 expression were significantly enriched for terms “oxidative phosphorylation” and “NADH dehydrogenase complex” (Fig. 2d). These data suggest that AMLs with the highest PDK1 expression display increased stemness signatures and adopt a more glycolytic metabolic state, while AMLs with low PDK1 expression display a more OXPHOS-driven program, which we could functionally confirm in Seahorse studies (Fig. 2e). Another striking feature that emerged from our GSEA studies was the observation that AMLs with low PDK1 expression (PDK1^low^ AMLs) were strongly enriched for FLT3-ITD signatures and that PDK1^high^ AMLs were enriched for signatures that were downregulated in NPMcyt AMLs (Fig. 2d). Indeed, we could confirm that PDK1 expression was significantly higher in FLT3-wt and NPM-wt AMLs (Fig. 2f). Moreover, FLT3-wt AMLs displayed a significantly enhanced metabolic abundance of hexoses, whereby glucose is also included as a hexose sugar (Fig. 2g).

**Fig. 3:**
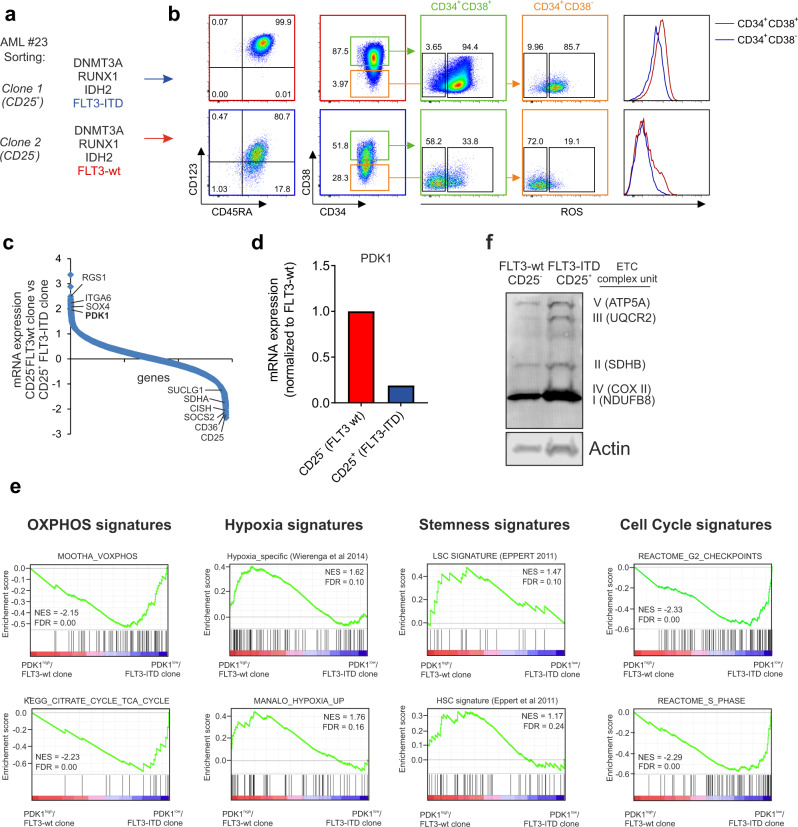
Subclone-specific metabolic differences in an individual patient. **a** Two genetically distinct subclones could be identified and prospectively sorted on the basis of differential CD25 expression (based on ref. ^7^). **b** ROS levels were low in the CD34^+^/CD25^−^ FLT3-wt subclone 2 and high in the CD34^+^/CD25^+^ FLT3-ITD subclone 1. **c** RNAseq of the CD34^+^/CD25^−^ FLT3-wt subclone was compared to the CD34^+^/CD25^+^ FLT3-ITD subclone. **d**, *PDK1* mRNA levels were highest in the CD34^+^/CD25^−^ FLT3-wt subclone. **e** Gene set enrichment studies performed on the ranked gene list depicted in **c**. **f** Western blot analysis of ETC Complex subunits; Complex I (NDUFB8, 18 kD), II (SDHB, 29 kD), III (UQCRC2, 48 kD), IV (COX II, 22 kD) and V (ATP5A, 54 kD) in FLT3-ITD mutant and FLT3-wt AML subclones (whole-cell lysates). Equal amount of protein loaded from both subclones and Actin used as the loading control.

Differences in metabolic signatures were further investigated in a patient in which we previously demonstrated that genetically distinct clones, differing in FLT3 mutation status, could be identified and prospectively sorted on the basis of differential CD25 expression (ref. ^7^ and Fig. 3a). The CD34^+^/CD25^+^ FLT3-ITD cells (clone 1) were high in CD34^+^/CD38^+^ and strongly positive for CD123 and CD45RA, associated with a L-GMP phenotype, while the CD34^+^/CD25^−^ FLT3-wt subclone (clone 2) was more immature with a high proportion of CD34^+^/CD38^−^ cells (Fig. 3b). In line with observations described above, ROS levels were much lower in the CD34^+^/CD25^−^ FLT3-wt subclone compared to the FLT3-ITD subclone (Fig. 3b). These differences were seen in both the CD34^+^/CD38^+^ fraction as well as the presumed LSC-enriched CD34^+^/CD38^−^ fraction. We ranked gene expression profiles of the 2 subclones and noted that *PDK1* scored among the top differentially expressed genes (Fig. 3c, d). Reversely, the FLT3-ITD subclone expressed high levels of CD25 as expected, but also high levels of SDH and SUCGL, and CD36 (Fig. 3c). Gene set enrichment studies further highlighted that OXPHOS and cell cycle related signatures were significantly enriched in the PDK1^low^ FLT3-ITD subclone, while hypoxia and stemness signatures were enriched in the PDK1^high^ FLT3-wt subclone (Fig. 3e). Western blotting confirmed an increase in ETC complex I-V protein expression in the FLT3-ITD subclone (Fig. 3f). We made sure to load equal protein amounts but noted that actin levels were also consistently slightly higher in the CD34^+^/CD25^+^ FLT3-ITD clone, possibly in line with the observation that these cells were also larger in size. These data further underline the heterogeneity of AML and show that metabolic profiles can even differ between genetically distinct subclones found in individual patients.

### AML subtypes display distinct metabolic dependencies and PDK1 governs metabolic activity in AML

**Fig. 4:**
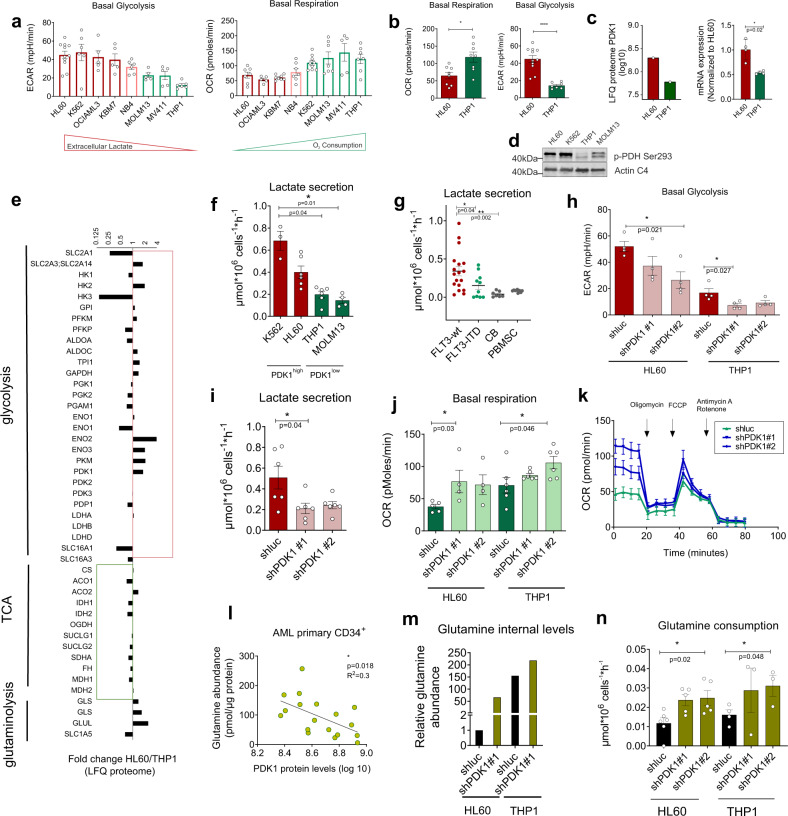
AML subtypes demonstrate different metabolic dependencies and PDK1 regulates metabolic response in AML. **a** ECAR and OCR (Seahorse assay) in eight AML cell lines (mean ± SEM, each dot represents independent replicates as mean of quadruplicate measurements). **b** Basal ECAR, OCR levels in THP1 and HL60 cells (*p = 0.014,****p < 0.0001). Each dot represents (*n* = 8) biological replicates (mean of quadruplicates) (mean ± SEM). **c**, PDK1 protein (log10) (*n* = 1) and relative mRNA expression in HL60 and THP1 cells. Each dot represents (*n* = 4) independent replicates (mean of quadruplicate measurements) (mean ± SD). **d** Western blot for P-PDH Ser293 and Actin (representative image of three independent experiments). **e** Protein expression of glycolysis (red), TCA cycle (green) and glutaminolysis related enzymes in HL60 *versus* THP1 cells. **f** Lactate secretion per 1 × 10^6^ cells per hour in K562, HL60, THP1, and MOLM13 cells (each dot represents biological replicates (mean of technical quadruplicates) and in **g**, CD34^+^ primary FLT3-wt (*n* = 7, in triplicates, duplicates), FLT3-ITD^+^ (*n* = 4, in triplicates) patient samples and Cord Blood (*n* = 3, in triplicates, duplicates) and PBMSCs (*n* = 3, in triplicates). **h** Basal ECAR in GFP^+^-sorted shLuc, shPDK1#1, shPDK1#2 THP1 and HL60 cells (each dot represents biological replicates (mean of quadruplicates). **i** Extracellular lactate concentration per 1 × 10^6^ cells per hour in HL60 shLuc, shPDK1#1, shPDK1#2 cells (*n* = 6 biological replicates, in triplicates). **j** Basal OCR in GFP^+^-sorted shLuc, shPDK1#1, shPDK1#2 THP1 and HL60 cells (*n* = 4, 5 biological replicates, measured in quadruplicates). **k** Real-time OCR in HL60 shLuc, shPDK1#1, shPDK1#2 GFP^+^-sorted cells upon Oligomycin A, FCCP and Antimycin A and Rotenone injections. **l** Pearson correlation of internal glutamine levels versus PDK1 protein expression in AML primary CD34^+^ samples (*n* = 18) (*p* = 0.018). **m** Intracellular glutamine levels in GFP^+^-sorted shLuc, shPDK1#1, shPDK1#2 THP1 and HL60 cells. Intracellular glutamine concentration was corrected to internal standards, and normalized to the protein amount. The glutamine abundance in shluc HL60 cells was set to 1. **n** Glutamine-consumption levels per 1 × 10^6^ cells per hour in culture medium in GFP^+^-sorted shLuc, shPDK1#1, shPDK1#2 THP1 and HL60 cells (each dot represents biological replicates (in technical quadruplicates) (mean ± SEM). **b**, **c** Student’s *t*-test (two-sided) or **f**, **g**, **h**, **i**, **j**, **n** Kruskal–Wallis one-way ANOVA test for multiple comparisons or (l) linear regression analysis.

To further functionally investigate the heterogeneous metabolic profiles seen in primary AML patient samples we screened 8 AML cell lines using various approaches. Like in patients, AML cell lines differed considerably in their metabolic profiles, whereby HL60 cells were among the most glycolytic with high extracellular acidification rates (ECAR) and low oxygen consumption rates (OCR), while THP1 cells (MLL-AF9^+^) and FLT3-ITD-positive AML cells (MOLM13 and MV411) were predominantly OXPHOS-driven (Fig. 4a, b). PDK1 protein and mRNA expression were higher in HL60 in comparison to more OXPHOS-dependent THP1 cells (Fig. 4c). Pyruvate Dehydrogenase (PDH) can be phosphorylated at serine residues on its E1α subunit by PDKs^36,37^. Thus, as an independent validation to check PDK1 activity, P-PDH Ser293 levels were analyzed by Western blot. P-PDH Ser293 was remarkably higher in both HL60 (PDK1^High^) and K562 cells (PDK1^high^) and lower in both MOLM13 and THP1 cells (PDK1^Low^) (Fig. 4d). In addition, in line with our primary AML patient data, a full quantitative proteome analysis revealed that TCA-related enzymes were higher expressed in THP1 cells while glycolysis-related enzymes, including PDK1, were highest in glycolytic HL60 cells (Fig. 4e).

These functional Seahorse data were then independently confirmed using spectrophotometric enzymatic assays. Similar to higher lactate efflux activity in glycolytic PDK1^High^ HL60 and K562 cell lines, FLT3-wt AML primary CD34^+^ cells were notably higher in their glycolytic lactate secretion activity compared to FLT3-ITD AML cell lines and primary CD34^+^ cells and healthy CB and PBSC CD34^+^ counterparts (Fig. 4f, g). To functionally asses the role of PDK1, we used PDK1-directed shRNAs to suppress PDK1 activity (Supplementary Fig. 3a, b). Downregulation of PDK1 expression led to a reduction in basal glycolysis (Fig. 4h), lactate secretion (Fig. 4i) and a concomitant increase in basal respiration (Fig. 4j, k) in HL60 cells. While glycolysis was already lower at baseline in the OXPHOS-driven THP1 cells, this could be further reduced upon knockdown of PDK1. Similarly, basal respiration could be further enhanced (Fig. 4j). Knockdown of PDK1 in the NB4 cell line (displaying intermediate glycolysis and mitochondrial OXPHOS) also still impacted on their glycolytic activity towards increased respiration (Supplementary Fig. 3c). These data indicate that PDK1 acts as the gatekeeper of the glycolytic state in these cells. To evaluate PDK1 specificity, we performed shRNA knockdown experiments on PDK2 and PDK3 in HL60 cells, but the effects on the glycolytic and mitochondrial respiration states were much less pronounced compared to knockdown of PDK1 (Supplementary Fig. 3d). We also used the glycolysis inhibitor 2-DG (2-deoxy-D-glucose), and while ECAR levels were significantly reduced in HL60 cells after 24 hrs treatment, no effects were observed on OCR (Supplementary Fig. 3e).

As PDK1 protein expression was negatively correlated with intracellular glutamine levels in primary AML patients (Fig. 4l) and *PDK1* mRNA levels were negatively correlated with glutamine levels in AML cell lines (*n* = 12) in the CCLE metabolome dataset (Supplementary Fig. 3f), we speculated that OXPHOS-driven cells would ensure a higher uptake of glutamine to use this as a carbon source to maintain Krebs cycle activity. Indeed, we observed that at baseline THP1 cells have much higher levels of intracellular glutamine compared to glycolytic HL60 cells, but that knockdown of PDK1, thereby driving cells from glycolysis into OXPHOS, also coincides with a strong increase in intracellular glutamine levels (Fig. 4m). To independently confirm these findings we monitored glutamine metabolism using LC-MS/MS to detect extracellular glutamine levels in the culture medium and we used spectrophotometric enzymatic assays to determine glutamine consumption per cell. Thus, we identified that glutamine consumption was higher in THP1 cells compared to HL60 cells, which could be further increased upon PDK1 knockdown (Fig. 4n).

### PDK1 inhibition decreased leukemic tumor growth in vitro and in vivo

**Fig. 5:**
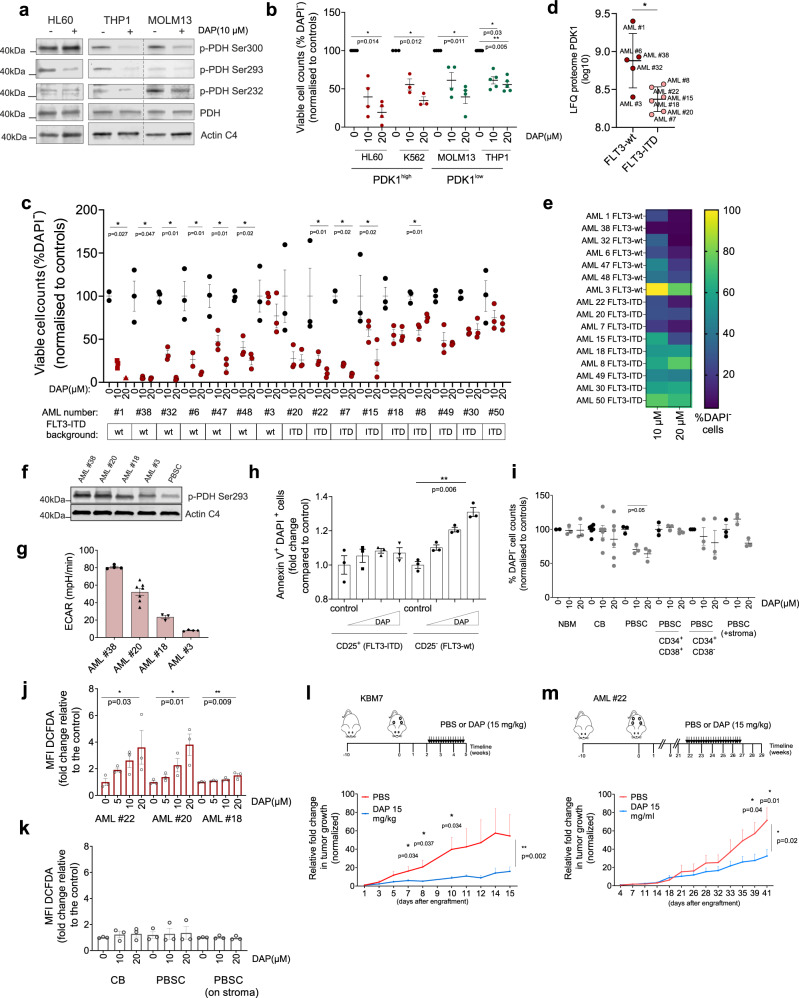
Pharmacological inhibition of PDK1 reduces leukemia growth in vitro and in vivo in humanized niche xenograft mouse models. **a** Western blot analysis of PDH-subunits with/without 10 μM DAP (24 h) in AML cells (a representative of two independent replicates). **b** (%) DAPI^−^ viable AML cells upon 10, 20 μM DAP (24 h) (HL60 and MOLM13: *n* = 4; K562: *n* = 3; THP1: *n* = 5, each dot shows means of technical triplicates). **c** as in **b**, but shows AML primary CD34^+^ cells (*n* = 16, dots show technical triplicates) with DAP (3 days) on MS5-stroma. Data was normalized to the controls in panel **b**, **c** (mean ± SEM). **d** PDK1 protein expression (log10) in FLT3-ITD^+^ (*n* = 5) and FLT3-wt (*n* = 6) AML primary CD34^+^ cells from panel **c** (mean ± SD, *p* = 0.030). **e** Heatmap demonstrating DAP sensitivity in FLT3-ITD + (*n* = 9), FLT3-wt (*n* = 7) patient samples from panel **c**. **f** Western blot analysis of P-PDH Ser293 in PBMSC (*n* = 1), in selected AMLs (*n* = 4) from panel **c** (a representative of two independent replicates). **g** ECAR in CD34^+^ AML patient samples (*n* = 4 patients, dots show technical replicates) from panel **c**, **f**. Equal viable cell numbers were screened. **h** Fold-change in Annexin V^+^/DAPI^+^ cells CD25^+^/FLT3-ITD^+^ and CD25^−^/FLT3-wt subclones with/without increasing concentrations of DAP (48 h) (data was normalized to controls, *n* = 3 technical replicates shown as representative of the two biological repeats). **i** Percentage DAPI^−^/CD34^+^ NBM (*n* = 1, technical triplicates), CB cells (*n* = 6, each dot shows mean of technical triplicates) and PBSCs (sorting as indicated) upon 10, 20 μM DAP (3 days) (*n* = 3, in technical triplicates in each). **j** Mean fluorescent intensity of DCFH-DA (ROS) after 3 days DAP treatment (10, 20 μM) in AML primary cells (*n* = 3, measured in technical triplicates) and **k**, in healthy CB CD34^+^ (*n* = 2, measured in technical triplicates), CD34^+^ PBSC cells (*n* = 3 for liquid, *n* = 2 for MS5-stroma co-cultured). **l** In vivo experimental set-up for (intrascaffold) KBM7-transplanted (*n* = 20, 3 scaffolds per mouse) and m, for AML primary CD34^+^-transplanted mice (*n* = 20, 4 scaffolds per mouse). Black arrows indicate treatment days. Relative fold-changes in the tumor sizes compared to the start of the treatment in control and DAP (15 mg/kg) (mean ± SEM). **d**, **l**, **m** Student’s *t*-test (two-sided); **b**, **c**, **d**, **h**, **i**, **j**, **k** one-way ANOVA Kruskal–Wallis test for multiple comparisons.

As a clinically more relevant way to inhibit PDK1 activity we included the validated PDK1 inhibitor DAP (2,2-dichloroacetophenone)^38^ in our studies and examined its effects on proliferation, apoptosis and leukemia development in vitro and in vivo in humanized niche xenograft mouse models. Serine phosphorylation of the PDK1 target PDH was high in HL60 compared to THP1 and MOLM13 cells, which was reduced upon treatment with 10 μM DAP (Fig. 5a). Within 24 h strong effects were seen on AML cell lines upon PDK1 inhibition whereby cell viability was most strongly decreased in glycolytic HL60 cells compared to more OXPHOS-driven THP1 and MOLM13 cells (Fig. 5b). Reduced viability was associated with a strong induction of apoptosis as determined by flow cytometry/Annexin V-DAPI staining (Supplementary Fig. 3g). Lactate secretion was strongly reduced upon DAP treatment (Supplementary Fig. 3h). More importantly, we also treated a large panel of primary patient samples (*n* = 16) grown on bone marrow stromal co-culture conditions and compared their PDK1 protein levels (in 11 out of 16) and ITD mutational backgrounds (in all 16) to their sensitivity towards DAP. While heterogeneity in responses was noted, a clear trend towards greater sensitivity towards DAP was observed in FLT3-wt (mostly PDK1^High^) AMLs (Fig. 5c, e, f). Next, we evaluated sensitivity against OXPHOS-related inhibitors in comparison to PDK1 dependency of two FLT3-wt AMLs. We used the electron transport chain (ETC) complex I inhibitor Rotenone and ETC complex II inhibitors TTFA (thenoyltrifluoroacetone) and 3-Nitropropionic acid (as 3-NPA). While FLT3-wt CD34^+^ AML cells were very sensitive to DAP treatment, the impact of ETC inhibitors on cell viability was much less pronounced (Supplementary Fig. 3i). PDK1 inhibitor sensitivity was seen in both the CD34^+^/CD38^+^ fraction as well as in the presumed LSC-enriched CD34^+^/CD38^−^ sorted populations (Supplementary Fig. 3j). Starting with an unsorted approach using mononuclear AML blasts, we also observed comparable apoptosis levels after 3 days of DAP treatment in CD34^+^/CD38^+^ and CD34^+^/CD38^−^ fractions, while no apoptosis was induced in healthy HSCs or progenitors (Supplementary Fig. 3k). Additionally, we analyzed p-PDH Ser 293 protein levels as a PDK1 activity indicator and glycolytic activity of a few AMLs for which sufficient material was available. AML#38 (PDK1^High^, FLT3-wt) was highly sensitive to DAP and displayed high p-PDH Ser293 levels (Fig. 5f) and high basal glycolysis (Fig. 5g). AML#3 and AML#18 were low in PDK1 expression (Fig. 5d), low in PDK1 activity based on P-PDH Ser293 levels (Fig. 5f), low in basal glycolytic activity (Fig. 5g) and these AMLs were less sensitive to DAP (Fig. 5c). Despite the notion that FLT3-ITDs AMLs were generally low in PDK1 expression and less sensitive to DAP treatment (Fig. 5c–e), AML#20 provided an exception in that p-PDH Ser293 as well as ECAR levels were relatively high in this FLT3-ITD^+^ AML (Fig. 5f, g), which nicely correlated with DAP sensitivity (Fig. 5c). Finally, we were able to study DAP sensitivity in the context of two genetically distinct subclones within an individual patient, one clone carrying the FLT3-ITD and the other being FLT3-wt (described in Fig. 3). FLT3-wt subclones were considerably more sensitive to increasing concentrations of DAP, whereas the viability of the FLT-ITD subclone was not affected (Fig. 5h).

In order to screen for a potential therapeutic window a panel of control CD34^+^ cells from normal bone marrow (NBM), PBSC and CB were also treated for 3 days with DAP, but PDK1 inhibition did not strongly impair cell viability, not even of the most immature CD34^+^/CD38^−^ stem cell fraction (Fig. 5i). No differences in PDK1 inhibitor sensitivity were seen when PBSCs were grown in liquid culture conditions or on bone marrow stromal co-culture conditions (Fig. 5i). DAP treatment resulted in an increase in ROS levels in AML cell lines and AML primary samples (Fig. 5j and Supplementary Fig. 3i, which was again not observed in healthy CD34^+^ cells (Fig. 5k). The increase in ROS in AML cells treated with PDK1 inhibitor could be rescued by treatment with the antioxidant N-acetyl-cysteine (Supplementary Fig. 3l). Moreover, in the presence of NAC, the cell viability of AML cells upon PDK1 inhibition was also rescued (Supplementary Fig. 3m).

We further studied effects of DAP treatment on glycolysis-driven AML development and survival in vivo using our humanized niche scaffold-based xenograft models^39^. First, we used the haploid line KBM7 that was glycolytic and metabolically similar to HL60 cells (Fig. 4a), expressed high P-PDH293 (indicative of high PDK1) and high PDK1 (Supplementary Fig. 3n, o, respectively) and efficiently engrafts in mice equipped with MSC-coated scaffolds that express human IL-3 and TPO^40^. Within 5 weeks after intrascaffold injection, a lethal leukemia developed from these scaffolds, but treatment with the PDK1 inhibitor significantly reduced tumor growth in these mice (Fig. 5l). While almost 90% of the injected scaffolds developed tumors in the control group, less than 60% of the injected scaffolds developed tumors in DAP-treated animals (Supplementary Fig. 3p). Efficacy of PDK1 inhibitors was also evaluated in a primary patient-derived xenograft scaffold mouse model, for which we took AML #22 that we previously extensively analyzed in this model^39^, which was shown to be sensitive to DAP in vitro, and was characterized as a relatively glycolytic AML based on Seahorse data (Supplementary Fig. 3q). As shown in Fig. 5m, treatment with DAP significantly reduced tumor growth in this PDX huScaffold model as well.

### PDK1 inhibition sensitizes to inhibition of glutaminolysis

**Fig. 6:**
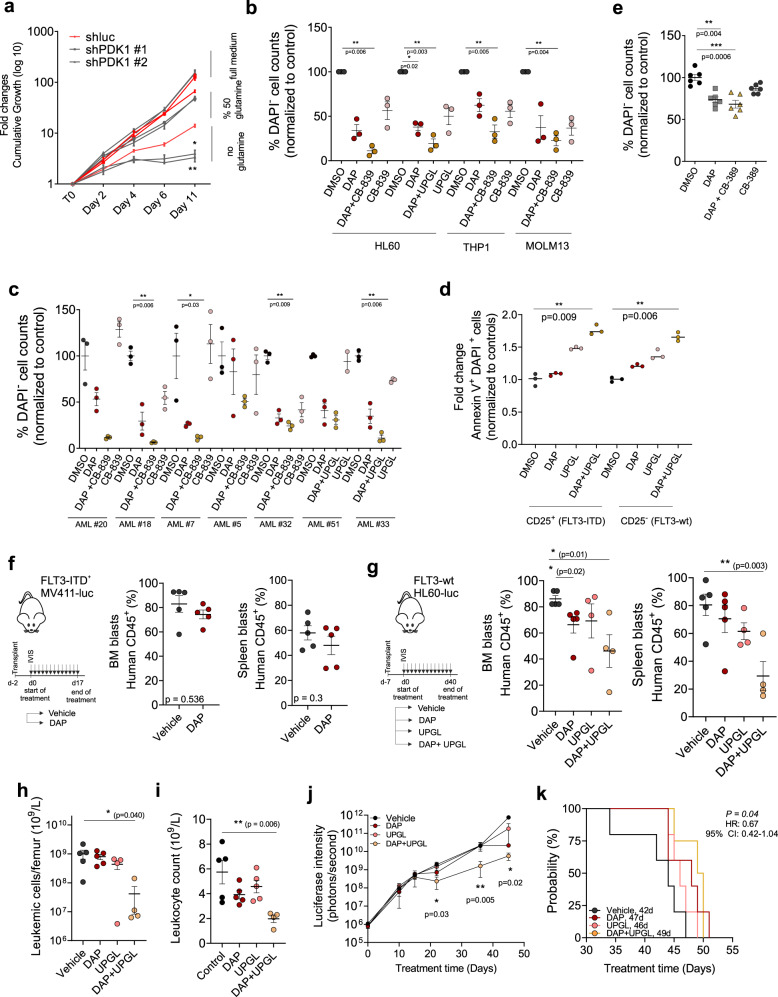
PDK1 inhibition metabolically sensitizes leukemic cells to GLS inhibition in vitro and in vivo. **a** Cumulative cell growth of GFP-sorted shLuc, shPDK1#1, shPDK1#2 THP1 cells in the presence (2 mM, 1 mM) and absence of glutamine (three independent experiments). (*p* = 0.04 for shPDK1#1 vs shluc, *p* = 0.0055 for shPDK1#1 vs shluc at d11) **b** (%) DAPI^−^ viable AML cells (*n* = 3 independently, as mean of technical replicates, 24 h) and **c** AML primary CD34^+^ cells (*n* = 7, in technical triplicates, 3 days on MS5-stromal co-culture) upon 10 μM DAP and/or 0.5 μM CB-839 and/or 0.5 μM UPGL00004 (UPGL). Data was normalized to controls. **d** Fold-change in Annexin V^+^/DAPI^+^ cells after treating CD25^+^-FLT3-ITD^+^ and CD25^−^-FLT3-wt sorted-subclones with 5 μM DAP and/or 0.5 μM UPGL (48 h). Data was normalized to controls, *n* = 3 technical replicates of one of the two independent experiments. **e** (%) DAPI^−^ Cord Blood (CB) CD34^+^ cells (*n* = 5 mean of technical triplicates) upon 10 μM DAP and/or 0.5 μM CB-839 (2 days). **f** Mice transplanted with MV411-luc cells were daily treated intraperitoneally with vehicle or DAP (20 mg/kg) (*n* = 5, in each group). Bar graphs demonstrate (%) human CD45^+^ cells in the bone marrow and spleen at the time of sacrifice (mean ± SEM). **g** Mice transplanted with HL60-luc cells were daily treated intraperitoneally with vehicle or DAP (20 mg/kg) or UPGL (3 mg/kg) or with combination of DAP and UPGL (*n* = 5 or 4, in each group). Bar graphs demonstrate (%) human CD45^+^ cells in the bone marrow and spleen at the time of sacrifice (mean ± SEM). **h** Total leukemic cell counts per femur and **i** total leukocyte counts in HL60-mice in each treatment group (mean ± SEM). **j** Luciferase intensity-analysis in HL60-transplanted mice in each treatment group. *x*-axis indicates treatment days, *y*-axis shows the luciferase-intensity (photons/second) (mean ± SEM). *p*-values indicate the difference between vehicle and combined treatment group (Tukey’s post hoc test). **k** Kaplan–Meier curve showing survival of HL60-transplanted mice treated with the indicated therapy. *p*-value (Log-rank test) indicate significance for comparisons of combination of DAP and UPGL treated and control mice. **f** Student’s *t*-test or **b**, **c**, **d**, **e**, **g**, **h**, **i** one-way ANOVA Kruskal–Wallis test or **a** two-way ANOVA for multiple comparisons.

Our data clearly indicate that AMLs characterized by high PDK1 expression are enriched for stem cell signatures, are relatively glycolytic, and that inhibition of PDK1 drives cells into an OXPHOS state coinciding with ROS-dependent cytotoxicity. Yet, we did not observe a complete response in vivo when mice were treated with PDK1 inhibitors as a single agent, and also in vitro we observed heterogeneity in responses. Therefore, we questioned whether we could identify additional vulnerabilities and synthetic lethal interactions together with PDK1 inhibition in AML cells. We reasoned that when cells would be released into an OXPHOS state upon inhibition of PDK1, their dependence on glutamine would increase in order to feed the TCA cycle. We first tested this hypothesis by downregulating PDK1 in THP1 cells followed by culturing under limiting levels of extracellular glutamine. In the presence of 1 mM Glutamine (50% reduction of in vitro physiological condition) a reduction in cell proliferation was observed, which was significantly lower in PDK1 knockdown cells, and this difference was even further pronounced when cells were grown without extracellular glutamine (Fig. 6a). Glutaminolysis can also be repressed with chemical compounds CB-839 and UPGL00004 (indicated as UPGL) that inhibit Glutaminase (GLS) activity, thereby preventing the conversion of glutamine into glutamate. We initially started our experiments with CB-839 but to be able to administrate intraperitoneal injection for our in vivo studies^41^ we included UPGL as well. Ultimately, the in vitro efficacy of both inhibitors appeared to be very comparable as outlined below. While modest responses were seen across a panel of AML cell lines upon treatment with only DAP or GLS inhibition as single agents at intermediate concentrations, combination treatment resulted in strong reductions in viability (Fig. 6b). These effects were even more pronounced in some cases when the combination treatment was evaluated on primary AML patient samples (Fig. 6c). The FLT3-ITD clone of AML patient #23 that was not sensitive to DAP treatment could be sensitized by co-treatment with glutaminolysis inhibitor (Fig. 6d). Stromal cells did not appear to be affected by this combination treatment (Supplementary Fig. 4a), suggesting that the bone marrow niche might tolerate such combination treatments. Healthy CD34^+^ cells were also not affected by the combination treatment compared to monotherapies (Fig. 6e).

After studying the in vivo effects of DAP PDX models of a highly glycolytic cell line and primary AML samples (Fig. 5l, m) we wished to compare the effect of in vivo PDK1 inhibition in PDK1^high^ (FLT-wt) and PDK1^low^ (FLT3-ITD) AML cell lines. To do so, we used luciferase-transduced HL60 cells (PDK1^high^, glycolysis^high^, OXPHOS^low^ Fig. 4) and MV4-11 and MOLM13 (PDK1^low^, glycolysis^low^, OXPHOS^high^ FLT3-ITD^+^, Fig. 4a and Supplementary Fig. 3j) models in vivo (Fig. 6f, g and Supplementary Fig. 4b, respectively). Overall survival analysis showed a modest (although statistically significant) effect of DAP in MV4-11 transplanted mice, which was not observed for the MOLM13 group (Supplementary Fig. 4c). As expected, a more pronounced effect of DAP monotherapy was observed for HL60-transplanted mice (Supplementary Fig. 4c). While bone marrow chimerism levels (measured by percentage of human CD45^+^ cells) were significantly lower after DAP treatment in HL60 transduced mice (Fig. 6g), disease burden in bone marrow and spleen was not impacted in both MV4-11 and MOLM13 transplanted mice (Fig. 6f and Supplementary Fig. 4b, respectively). We then compared the effect of mono- and combination therapy targeting PDK1 and GLS inhibition in HL60-transplanted mice. Mice were randomly distributed into the different treatment groups (Vehicle, DAP, UPGL and combination) based on luciferase intensity measured a week after the transplantation (Supplementary Fig. 4d). We observed that the combination treatment of DAP together with UPGL further reduced chimerism levels in the BM and spleen (Fig. 6g), as well as tumor load in the BM (Fig. 6h) of HL60 mice. Spleen weight and size were significantly reduced after double treatment compared to the control group (Supplementary Fig. 4e). Peripheral blood leukocyte counts at day 28 for each treatment showed a significant decrease in the combination treatment group in comparison to control mice (Fig. 6i). Tumor burden was significantly less pronounced over time in HL60 mice after combination treatment with DAP and UPGL compared to vehicle and single therapy regimens (Fig. 6j and Supplementary Fig. 4f). Lactate levels were decreased in the plasma of mice receiving combination treatment, suggesting reduced lactate efflux (Supplementary Fig. 4g). Inhibition of both PDK1 and GLS significantly prolonged survival of HL60-transplanted mice as compared with the vehicle treatment (Fig. 6k). Together, these data indicate that PDK1 inhibition sensitizes to inhibition of glutaminolysis, which should be exploited further clinically in the context of targetable metabolic rewiring capacity in AML.

## Discussion

The genetically heterogeneous landscape of AML significantly complicates therapy and warrants personalized treatment options. We used a comprehensive multi-omics approach in which we quantified the metabolome, proteome and transcriptome of a large cohort of primary human AML patient samples and compared those to healthy CD34^+^ hematopoietic stem/progenitor cells. These studies revealed that human leukemias display distinct metabolic states that can serve as targets for treatment. PDK1 was identified as such a determinant of different metabolic states, whereby PDK1^high^ AMLs are low in mitochondrial OXPHOS, are often wild type for FLT3 and NPM1, and are enriched for stemness signatures. On the other hand, AMLs with low PDK1 frequently carry the FLT3-ITD mutation, express high cell cycle, OXPHOS and L-GMP signatures, and display the highest oxygen consumption rates. Even within an individual leukemia patient that harbors genetically distinct subclones we observed clear metabolic differences. These observations further add to the notion that AML is a rather heterogeneous disease whereby even at the subclonal level within individual patients metabolic differences are observed.

In healthy mouse HSCs Pdks have emerged as important regulators of glycolysis, thereby controlling their quiescent state^29^. Pdks were highest expressed in the most primitive long-term HSCs, coinciding with increased levels of ser293 and ser300 phosphorylation of PDH, thereby rendering it in an inactive state and preventing pyruvate from entering the TCA cycle^29^. These data suggest that LT-HSCs can survive independent of the production of mitochondrial energy, in line with the notion that mitochondria in HSCs are relatively inactive^19^, although it was shown that a functional respiratory chain is essential for HSC differentiation^28^. Mitochondrial activity can be reduced by the NAD^+^-boosting agent nicotinamide riboside^16^, by using the mitochondrial respiratory chain uncoupler FCCP^15^, by depleting HSCs of the PTEN-like mitochondrial phosphatase PTPMT1^17^, or mitochondrial clearance^18^, which enhances self-renewal of HSCs^15^. In human CD34^+^ leukemic stem/progenitor cells, we find that PDK1 was most consistently upregulated across multiple independent proteome and transcriptome datasets, compared to normal CD34^+^ cells. The upregulation of PDK1 was not uniformly detected in all patients, but in particular in those that were enriched for LSC-signatures, coinciding with the lowest oxygen consumption rates. These AMLs were also the most sensitive to PDK1 inhibition, which resulted in elevated ROS levels most likely related to enhanced mitochondrial activity due to a release into the TCA cycle. Although in our studies we have not seen any ROS-mediated toxicity upon PDK1 inhibition in normal stem/progenitor cells, too high levels of ROS also appear to be toxic to normal HSCs. Low levels of ROS are required in order to maximize their lifespan^21^, and also leukemic stem cells have been proposed to maintain a ROS^low^ state^22^. PDK1 inhibition was also shown to be effective in AML cells lines, linked to a reduction in BCL2 and BCL-XL expression, increased PARP and Caspase-mediated apoptosis and a loss of autophagy regulators^42^. Together, these data indicate that PDK1 is an attractive target in AML, at least in a subset of patients that displays low mitochondrial respiration activity.

Interestingly, AML patients with low PDK1 expression levels were particularly enriched for FLT3-ITDs and displayed L-GMP-like and OXPHOS gene signatures. Also, ROS levels are elevated in FLT3-ITD AMLs^23,43–45^. Recently, we identified that within an individual patient two genetically distinct subclones could be identified, one being FLT3-ITD and the other clone being FLT3-wt^7^. We further characterized the metabolic states of these subclones in detail and observed that ROS levels were much lower in the CD34^+^/CD25^−^ FLT3-wt subclone compared to the FLT3-ITD subclone. These differences were also noted at the transcriptome level, which indicated that OXPHOS and cell cycle related signatures were significantly enriched in the PDK1^low^ FLT3-ITD subclone, while hypoxia and stemness signatures were enriched in the PDK1^high^ FLT3-wt subclone. Indeed, PDK1 expression ranked among the top upregulated genes in the FLT3-wt clone compared to the FLT3-ITD clone. Previously, we identified PDK1 as a HIF target gene that is strongly upregulated under hypoxia^46^, further underlining that hypoxia signaling networks are operational in PDK1^high^ cells.

We observed that intracellular glutamine levels were lower in PDK1^high^ FLT3wt cells compared to FLT3-ITD AMLs. It was recently shown that inhibition of FLT3-ITDs creates an additional dependency on glutamine influx in order to maintain TCA activity^47,48^. In line, we also observed that a release into oxidative phosphorylation by PDK1 inhibition rendered AML cells more sensitive to glutaminolysis inhibitors, or to deprivation of extracellular glutamine. Although it remains difficult to determine the direction of the glutaminolysis flux based on steady state metabolite levels, it may indicate that these cells rely more on glutamine as an alternative carbon source to maintain their TCA cycle. Collectively, these studies point out the importance of glutaminolysis metabolism in the general metabolic plasticity capacity of AML upon therapeutical interventions.

Recent work indicated that leukemic stem cells might be sensitive to OXPHOS inhibition^24–27^. It was shown that distinct subtypes of AML are particularly sensitive to mubritinib, which targets electron chain complex I activity^49^. Addiction to complex I activity was strongly associated with the presence of *NPM1* and *FLT3*-ITD mutations, and mubritinib-treated cells switched from OXPHOS to glycolysis. Moreover Serine 293 of the PDH E1 subunit, phosphorylated by PDK1, was upregulated at phospho-protein level upon mubrinitib treatment, suggesting an important metabolic regulatory role of PDK1 in OXPHOS inhibition resistant AMLs. Thus, targeting PDK1 might be also necessary for eradicating resisting pre-OXPHOS^high^ post-Glycolysis^high^ LSCs. Our data showing that PDK1^high^ AMLs are frequently NPM1 and FLT3-wt, low in mitochondrial respiration, and high in hypoxia signatures, are directly in line with these observations and further underscore the heterogeneous landscape of AML, also at the metabolome level. Strikingly, we also find that PDK1^high^ AMLs are low in *HOXA*/*B* gene expression, while *ZNF521* and *KIRREL* are high, opposite to what was observed in mubritinib-sensitive samples, and also glutamic-oxaloacetic transaminase 1 (GOT1), which was identified as a synthetic lethal interaction with mubritinib treatment^49^, is negatively correlated with PDK1.

Our targeted metabolome screen in AML and healthy stem and progenitors involved five major metabolite families including sphingolipid family. C16:0 levels were shown to be the most distinct type among other sphingolipids and were significantly high in healthy HSCs compared to the progenitors^50^. We identify that C16:0 was the most striking top sphingolipid and significantly highly abundant in AML stem and progenitors compared to healthy counterparts, suggesting its importance in both healthy and malignant stemness.

In conclusion, our studies highlight that the heterogeneous landscape of AML is also reflected in their metabolic states. As such, the metabolome provides an attractive alternative means for targeting LSCs, which should be further exploited. We show that PDK1 is upregulated in a genetically distinct subset of AML patients characterized by LSC-enriched transcriptome signatures where it acts as a gatekeeper of the glycolysis-OXPHOS state. Targeting PDK1 drives AML cells into an oxygen consumption rate mode resulting in mitochondrial stress-mediated toxicity and PDK1 inhibition sensitizes leukemic cells to GLS inhibitors as a consequence of glutamine-dependent metabolic rewiring.

## Methods

### AML and healthy primary cell isolations and in vitro cell culture conditions

Human myeloid cell lines used in this study were maintained in RPMI 1640 medium supplemented with 10% fetal calf serum (FCS) (Sigma Aldrich), 1% penicillin and 1% streptomycin (Life Technologies, Grand Island, USA) and 2 mM Glutamine at 37 °C and 5% CO_2_.

Neonatal cord blood (CB) samples were obtained from healthy full-term pregnancies and AML specimens were obtained from apheresis product, peripheral blood, or bone marrow from AML patients and mobilized peripheral blood from healthy donors who gave informed consent about procedures were obtained in accordance with the Declaration of Helsinki at the obstetrics departments at the Martini Hospital and University Medical Center Groningen. The study was approved by the UMCG Medical Ethical Committee. Mononuclear cells (MNCs) were isolated via Lymphoprep^TM^ (Alere technologies, Oslo, Norway) density gradient-based separation and either used fresh or cryopreserved in our biobank until further use. When cryopreserved MNC fractions of AML patients and PB from allogeneic donors were used, samples were thawed and then firstly resuspended in newborn calf serum (NCS) supplemented with DNase I (20 Units/mL), 4 mM MgSO4 and heparin (5 Units/mL) and incubated on 37 °C for 15 min (min). Next, CD34^+^ cells were isolated using autoMACS hematopoietic progenitor magnetic-associated cell-sorting kit automatically from Miltenyi Biotech according to the manufacturer’s instructions.

Primary AML cells were plated in Gartner’s medium consisting of α-MEM (Thermo Scientific) supplemented with 12.5% heat-inactivated fetal calf serum (Sigma Aldrich), 12.5% heat-inactivated horse serum (Invitrogen), 1% penicillin and streptomycin (Life Technologies, Grand Island, USA), 57.2 µM β-mercaptoethanol (Merck Sharp & Dohme BV) and 1 mM hydrocortisone (Sigma-Aldrich), with the addition of 20 ng/mL G-CSF (Amgen), N-Plate (Clinical grade TPO) (Amgen) and IL-3 (Sandoz). For primary AML co-cultures, mouse bone marrow derived MS5-stromal cells were initially plated on gelatin coated culture flasks and expanded to form a confluent layer, to which primary AML cells were added. Liquid cultures and co-cultures were grown at 37 °C and 5% CO_2_. Images of mouse bone marrow derived MS5-stroma cells were acquired on Leica DMIL inverted phase microscope (Leica Microsystems, Eindhoven, Netherlands).

Healthy NBM, CB, and peripheral blood mononuclear-stem cells (PBMSC) were cultured in rich Stem line-S0192 (Sigma Aldrich) medium with 1% penicillin and streptomycin containing cytokines c-Kit (100 ng/ml) (255-SC, Novus Biologicals), FLT3-Ligand (100 ng/ml), N-Plate (100 ng/ml) and IL-3 (100 ng/ml).

### In vivo assays using humanized scaffold nice xenograft models

The humanized niche scaffold NSG mouse model was established as described previously^39,51,52^ with addition of liquid extracellular matrix (Corning, 354230). Mouse experiments were performed in accordance with national and institutional guidelines. Scaffolds were transplanted subcutaneously into the flanks of 6- to 8-week-old female NOD.Cg-PrkdcscidIl2rgtm1Wjl/SzJ (NSG) mice. Scaffolds were injected with bone marrow derived mesenchymal stroma cells (BM-MSCs) of which 80% were wild type (wt) MSCs, 10% IL-3- and 10% TPO-expressing MSCs^40^. Six to eight weeks after scaffold implantation 1.5 × 10^6^ CD34^+^ isolated AML primary human cells and 1 × 10^6^ KBM7 leukemic cells were injected into either four scaffolds per mouse for the primary AML or three scaffolds per mouse in case of the leukemic cell line. One day before transplanting human cells into the humanized nice, mice were sub lethally irradiated (200 rad). Human CD45 engraftment was analyzed by timely submandibular bleeding procedures. Treatment was started when tumor volumes became palpable, mice were sacrificed when tumor volumes reached ethical limits or if mice showed severe signs of illness. 15 mg/kg DAP was injected intraperitoneally every day for KBM7-transplanted mice and every 5 days for 5 weeks for the AML primary transplanted mouse. Tumor size was measured every 3–4 days using a Vernier caliper and volume was calculated as cm^3^. Cells from the humanized scaffolds and mouse organs, including BM, spleen, and liver were isolated and analyzed by FACS.

### In vivo assays using MOLM13, MV411, and HL60 cells

For AML xenografic models, 8-week-old female NSG mice (005557, The Jackson Laboratory) were pre-conditioned with 20 mg per Kg of busulfan (Sigma-Aldrich) 1 day prior to the transplant. On the next day, 5 × 10^5^ luciferase-transduced MV4-11, HL60 and MOLM13 cells were intravenously injected, as indicated in the schematic for the murine model (Fig. 6f, g and Supplementary Fig. 4b, respectively). For measuring luciferase amount, mice were anesthetized and injected intraperitoneally with the firefly luciferase substrate D-luciferin (100 mg per Kg, PerkinElmer) and were then imaged with the IVIS Lumina system (Caliper Life Sciences Inc, Hopkinton, MA). Two days after the engraftment in MV411 transplanted mice and 1 day after the engraftment in MOLM13 transplanted mice, the mice were randomly assigned to two groups (*n* = 5 per group for MV411 mice according to IVIS results and *n* = 4 or 3 for MOLM13 mice) and treated with vehicle (PBS with 1%DMSO) and DAP (Sigma-aldrich; 20 mg per Kg; intraperitoneally). For HL60 mice, a week after transplant, the mice were randomly assigned to four groups (*n* = 4 to 5 per group) based on IVIS screening and treated with vehicle (PBS with 1% DMSO), DAP (Sigma-aldrich; 20 mg per Kg; intraperitoneally), UPGL00004 (indicated as UPGL) (Sigma-Aldrich; 3 mg per Kg; intraperitoneally) or a combination of these. The leukemic burden of HL60 cells was monitored by bioluminescence imaging at Day 0 and then weekly. Chimerism of MV4-11, MOLM13 and HL60 cells (evaluation of the percentage of human viable CD45^+^ cells) in the spleen, peripheral blood and bone marrow were evaluated by flow cytometry. Mice were followed for overall survival analysis and sacrificed when mice showed severe signs of illness. At the end of the experiment, animals were harvested and subjected to analysis of spleen and bone marrow. Only mice with viable material (bone marrow or spleen) were included in the analysis of bone marrow counts and chimerism. All animals were housed under specific pathogen free conditions in individually ventilated cages during the whole experiment and were maintained according to the Guide for Care and Use of Laboratory Animals of the National Research Council, USA, and to the National Council of Animal Experiment Control recommendations. All experiments were approved by the Animal Ethics Committee of the University of Sao Paulo and by Central Animal Facility University Medical Center Groningen (#067/2018).

### Mass spectrometry-based-targeted metabolomics assay

AbsoluteIDQ® p180 Kit (BIOCRATES Life Sciences AG, Innsbruck, Austria) was used for high-throughput metabolome screening. In all, 5–10 × 10^6^ cells from Primary AML, CB, PB samples (all primary samples processed after CD34^+^ isolation) and cell lines were washed three times with ice-cold PBS after centrifugation for 5 min at 300 × *g*, 0 °C, and cell pellets were snap-frozen in liquid nitrogen and stored at –80 °C until their analysis. The cell lysates to be loaded in the kit were prepared the day before of analysis. Thus, pellets were thawed on ice and then resuspended in 50 µL of 85:15 EtOH-PBS. This step was followed by ultra-sonication using a titanium probe for three times 15 s on ice, followed by snap freezing in liquid nitrogen for 30 s and fast thawing at 95 °C. This loop was repeated two times. Extracts were centrifuged at 16,000 × *g*, 5 min at 4 °C, and the cell lysate supernatants were frozen in liquid nitrogen and stored at –80 °C. The day of analysis, standards, internal standards, quality controls (10 μL of each) and cell lysate supernatants (30 to 50 μL) were loaded into the 96 well plate supplied with the AbsoluteIDQ p180 Kit, that was processed according to manufacturer instructions. LC-MS/MS (liquid chromatography-mass spectrometry) analysis (to detect amino acids and biogenic amines) and FIA (Flow Injection Analysis)-MS/MS analysis (to detect acylcarnitines, hexoses, glycerophospholipids and sphingolipids) were done using a MS/MS Sciex Triple Quadrupole 6500 Mass Spectrometer (AbSciex, Framingham, MA, USA) coupled to a 1290 Infinity LC (Agilent Technologies, Santa Clara, CA, USA). Total protein content in cell lysate supernatants was determined by Bicinconinic acid (BCA) assay (Thermo Fisher Scientific, Waltham, MA USA).

LC-MS/MS data was analyzed using Analyst 1.7 (AbSciex, Framingham, MA, USA), and the quantitative generated data was merged with the FIA-MS/MS raw data using the MetIDQ™ software package to obtain the concentrations for the 180 analyzed metabolites. The data was then normalized against protein concentrations and sample volume loaded in the plate.

### Oxygen consumption and extracellular acidification rate measurements

Oxygen consumption rate (OCR) and Extra Cellular Acidification Rate (ECAR) were measured using Seahorse XF96 analyzer (Seahorse Bioscience, Agilent, US) at 37 °C. For AML cell lines 100.000 cells and for primary PB and AML cells 200.000 cells were seeded per well ﻿in poly-l-lysine (Sigma-Aldrich) coated Seahorse XF96 plates in 180 µL XF Assay Medium (Modified DMEM, Seahorse Bioscience). For OCR measurements, XF Assay Medium was supplemented with 10 mM Glucose and 2.5 µM oligomycin A (Port A), 2.5 µM FCCP (carbonyl cyanide-4-(trifluorometh oxy) phenylhydrazone) (Port B) and 2 µM antimycin A together with 2 µM Rotenone (Port C) were sequentially injected in 25 µL volume to measure basal and maximal OCR levels (all reagents from Sigma-Aldrich). For ECAR measurements, Glucose-free XF Assay medium was added to the cells and 10 mM Glucose (Port A), 2.5 µM oligomycin A (Port B) and 100 mM 2-deoxy-D-glucose (Port C) (all reagents from Sigma-Aldrich). The XF96 protocol consisted of a four times mix (2 min) and measurement (2 min) cycles, allowing for determination of OCR/ECAR at basal and also in between injections. Both basal and maximal OCR levels as well as basal ECAR and glycolytic capacity measurements were calculated by assessing metabolic response of the cells in accordance with the manufacturer’s suggestions. The OCR and ECAR measurements were normalized to the number of cells used for the assay.

### Viability assays

Primary healthy and AML samples and AML cell lines were cultured (as described in the in vitro cell culture conditions’) with indicated medium and with and without inhibitors for 24 h or up to 3 days. Viability was assessed by DAPI staining and counted using a MACSQuant flow cytometer (Miltenyi Biotech) in a 96 wells format and analyzed by FlowJo v10.0.6 software (TreeStar, Ashland, OR). AML cell lines were used at 20.000 cells per well and NBM, CB, PBMSC cells were plated between 5000 and 9000 per well.

### Reagents

2, 2-dichloroacetophenone (DAP) was purchased from Sigma (St. Louis, MO). Stock solutions of the drug were prepared at 7 mM in dimethyl sulfoxide (DMSO). Stock solutions of CB-839 (Selleckchemicals, cat no: s7655) were prepared in DMSO at 87.95 mM. TTFA (2-Thenoyltrifluoroaceton, Cat# T27006), Rotenone (Cat# R8875), UPGL00004 (Cat#SML2472), 2-DG (Cat#D8375) and 3-Nitropropionic acid (Cat# N5636) were purchased from Sigma-Aldrich. Cells were treated from 24 h up to 3 days and then counted and analyzed by FACS.

### RNA isolation and qPCR

Total RNA was isolated using the RNeasy Mini Kit (QIAGEN) according to the manufacturer’s recommendations. For quantitative RT-PCR, RNA was reverse transcribed using the iScript cDNA synthesis kit (Bio-Rad) and amplified using SsoAdvanced SYBR Green Supermix (Bio-Rad) on a MyIQ thermocycler (Bio-Rad). Data was analyzed using the ΔΔCT relative quantification with MyIQ software (Bio-Rad). *RPL27* or *RPL30* was used as a housekeeping gene for normalization. Primer sequences are indicated in Key Resources Table.

### ShRNA design and lenti-viral transduction

Two different constitutive mir-E shRNA constructs targeting each human PDK1 were generated using 97-mer oligomers (from Sigma) PCR amplified and then cloned into to miR-E recipient vectors using XhoI/EcoRI restriction enzymes as described in detail previously^53^.

Short-hairpin RNA sequences used in this study are the first two shRNA sequences for each gene of interest listed in Zuber et al.^53^ except for the shluc. Shluc(firefly): CCGCCTGAAGTCTCTGATTAA; shPDK1 (#1): TTAGAGACTGTGTTGTTAGTTA; shPDK1 (#2): ACAGTGTAGTTATGAAAATATA; shPDK2 (#1): ATCCAGCAATGCCTGTGAGAAA; shPDK2 (#2): TCCAGCAATGCCTGTGAGAAAA shPDK3: ACAGGTCTTGGATAACTTTCTA. Lentiviruses containing a pRRL.SFFV.EGFP (gift from Christopher Baum) backbone, shRNA for PDK1 constructs, and as a control a pRRL.SFFV.EGFP vector containing a short-hairpin against renilla luciferase constructs were generated using the Fugene transfection system (Promega, Madison, USA), together with packaging construct psPAX2 (Addgene, #12260) and Glycoprotein envelope plasmid pMD2.G (Addgene, #12259). 293T cells cultured in DMEM (-FCS + 1% pen/strep) were transiently transfected in T75 flasks. Viral supernatant was harvested and filtered through a 0.45 µm filter after 2 days of the transfection and either used to transduce recipient cells (with 8 μg/mL polybrene to increase the infection efficiency) or stored in −80 °C for future usage. For transduction efficiency, GFP expression was checked using flow cytometry.

### Spectrophotometric enzyme activity assays

Both extracellular lactate and glutamine concentrations were determined from the cell culture medium by monitoring production or consumption rate of NAD(P)H by specific enzymatic reactions for each metabolite at 340 nm wavelength. Extracellular lactate concentrations were determined by the lactate dehydrogenase (LDH) enzymatic reaction in the cell culture medium taken at 0 h and after 24 h incubation. Extracellular lactate was converted by l-lactic dehydrogenase (LDH, Sigma Aldrich) reaction in freshly prepared 25 mM NAD^+^ and 87.7 U/mL LDH in 0.4 M hydrazine (Sigma Aldrich)/0.5 M glycine assay buffer (pH 9). 20 µL samples (diluted according to standard curve) and Sodium l-lactate (Sigma Aldrich) standards were pipetted into 130 µL reagent mix in 96 wells plate format and the reaction was carried out for 30 min at 37 °C. The concentration of extracellular glutamine was determined by its conversion first to glutamate through the glutaminase (GLS) reaction followed by the quantification of glutamate concentration. GLS reaction was carried out for 30 min at 37 °C with shaking by adding 20 µL sample of culture media to 180 µL reaction mix consisted of 10 U/mL GLS (Sigma Aldrich, Cat#G8880) in 0.5 M acetate buffer, pH 5. Determination of glutamate concentration was performed through the glutamate dehydrogenase (GLDH, Sigma Aldrich) reaction at 37 °C for half an hour by adding sample media to the reaction mix containing 25 mM ADP, 40 mM NAD^+^ and 100 U/mL of GLDH in 0.5 M glycine/0.5 M hydrazine buffer, pH 9. Glutamine concentration is calculated by deducting basal glutamate concentration from the final converted glutamate measurement. Multiskan Sky microplate reader (Thermo Fisher Scientific) spectrophotometer was used to detect the absorbance and the enzyme activity for each sample was normalized to the cell proliferation curve after 24 h.

### Flow cytometry and sorting procedures

Prior to antibody staining, cells were blocked with anti-human FcR Block (Mylteni Biotech) and murine cells were blocked with anti-Fc (BD Biosciences). For in vivo studies, tumor cells were stained with anti-CD34, anti-CD38, anti-CD45 (Biolegend), anti-CD33, anti-CD11b, anti-CD14, anti-CD15, anti CD-13, anti-CD3, anti-CD117 and anti-CD19 antibodies (all from BD Biosciences unless indicated otherwise) at 4 °C for 30 min followed by DAPI^−^ staining for 10 min. Samples washed twice in PBS before each flow-cytometric measurements. Fluorescence was measured on the MACSQuant Analyzer 10 (Miltenyi Biotech). Primary AML, Healthy CD34^+^ CB and PBMSCs cells were stained with anti-CD34 and anti-CD38 antibodies prior to the flow cytometric and sorting experiments.

A representative Flow-cytometry gating strategy for live cells and DAPI^−^ cells (after singlets) in primary cells, cell lines and healthy controls are indicated in Supplementary Fig. 3l. Apoptosis was quantified with Annexin V staining (FITC) according to manufacturer’s protocol (Mylteni Biotech). Equal number of cells are stained with Annexin V in calcium supplied sterile water buffer at a concentration of 1 × 10^6^ cells/mL for 20 min at 4 °C in dark followed by DAPI^−^ staining for 10 min.

For ROS measurements, 2′,7′-dichlorofluorescein diacetate (DCF-DA) (Sigma Aldrich) was dissolved as a 10 mM stock solution in DMSO. Cells were resuspended in PBS containing the probe at a 10 μM final concentration. Cells were stained for 30 min. at 37 °C in dark. Accumulation of DCF in cells was measured by an increase in fluorescence at 530 nm when the sample is excited at 485 nm (FITC channel). Fluorescence was measured on the MACSQuant Analyzer 10 (Miltenyi Biotech). ROS scavenger N-Acetyl-L-Cysteine (NAC) (Sigma Aldrich, Steinheim, Germany) was freshly prepared prior to each experiment and used in 2 mM final concentration in culture medium.

Cell sorting for AML primary stained samples, for CD3^−^CD34^+^CD25^+^ and CD3^−^CD34^+^CD25^−^ (Biolegend, Cat# 302606) AML subclones and for cell lines GFP^+^ isolation was performed on a MoFlo-Astrios (Beckman Coulter). Flow-cytometric analyses, cell counting and viability measurements were determined on either a LSR-II (BD Biosciences), BD Accuri C6 (Beckton Dickinson, Breda, the Netherlands) or MACSQuant (Miltenyi Biotech) flow cytometer. Within replicates of an experiment the same flow cytometer was used. All flow-cytometry data was analyzed using FlowJo v10.0.6 software (TreeStar, Ashland, OR).

### Western blotting

In all, 1 × 10^6^ AML cell lines were lysed in RIPA buffer and lysates were boiled at 100 °C r 10 min in Laemmli buffer after measuring protein concentration using Pierce BCA protein kit (Cat #23227). Equal amount of 20 μg was loaded to 4–15% mini Protean TGX precast gels for SDS-PAGE (Bio-rad Cat#4561085) and transferred with a Biorad Trans-blot Turbo transfer system to LF-PVDF membranes (Trans-blot Turbo RTA Transfer kit Cat#1704274). Membranes were blocked in Odyssey blocking buffer (Cat#927-40000) for 1 h and then incubated overnight at 4 °C with either of the following primary antibody mix; 1:1500 P-PDH Ser 232 (rabbit, Millipore AP1063), 1:1500 P-PDH Ser 300 (rabbit, Millipore, Calbiochem AP1064), 1:1500 P-PDH293 (Millipore, Calbiochem AP1062), 1:1000 PDH E1 alpha (mouse, Abcam, ab110330), 1:3000 Actin (mouse, Santa Cruz Biotechnology, SC-47778), 1:3000 Actin (rabbit, Cell Signaling Technology, 4970) or Oxphos cocktail 1:3000 (Thermo Fisher Scientific Cat#45-8199). Scanning of the membranes was performed with an Odyssey Clx scanner (Li-Cor Biosciences) after incubation with the following secondary antibodies (1:3000); Alexa Fluor 6 goat anti rabbit (Invitrogen, A21109) and IRDye 800CW donkey anti mouse (Li-Cor 926-32212).

### Proteome, transcriptome, and GO and GSEA studies

Proteome data were derived from our previously published study^7^ and is available at PRIDE under PXD030463. UMCG transcriptome data was derived from De Jonge et al.^34^ and transcriptome dataset was used from the TCGA Cancer Genome Atlas Research^3^ and from Bloodspot/MILE (GSE13159)^54^. GO-analysis was performed using the online GeneOntology tool^55,56^. GSEA v4.1.0 on pre-ranked gene lists was performed with respect to MSigDB genesets C2 and C5 GO biological processes (version 7.3). A significance cutoff of FDR *q*-values < 0.25 was used^57^.

### Quantification and statistical analyses

LC-MS/MS data analysis was performed using the MetIDQ^TM^ software package (MetIDQ™ Biocrates Life Sciences (www.biocrates.com) was used for analyzing Mass Spectrometry-Based-Targeted Metabolomics Assay generated data. Significance of correlations of metabolite abundance and protein expression and statistical significance related to metabolite abundance comparison between AML versus healthy and/or between AML individuals was determined by Student’s *t*-test or one-way ANOVA for multiple comparisons using Excel or Graphpad Prism 8 software. *p*-values are indicated in figure legends. ClustVis (BETA) web tool was used for clustering analysis of the metabolites (https://biit.cs.ut.ee/clustvis/). For GSEA analyses an FDR *q*-value < 0.25 was considered significant.

In vivo data daily comparison between normal and treated animals was calculated using by Student’s *t*-test using Excel and Graphpad Prism 8 and overall comparison was assessed using Mann–Whitney test with Graphpad Prism 8. For comparisons between the different groups of treated mice, Kruskal–Wallis test (with Dunn’s post hoc) was used to compare continuous variables. The cutoff used to define engraftment in the peripheral blood or bone marrow of the leukemic mice was the presence of human cells (CD45^+^) ≥ 0.1. Overall survival was plotted using Kaplan–Meier plots, using Cox proportional hazard regression to compare the differences between the curves, providing the hazard ratio (HR) and the 95% confidence interval (CI). All *p*-values were two-sided with a significance level of 0.05. All calculations were performed using statistical package for the social sciences (SPSS) 19.0 and GraphPad Prism 8 software. Corel draw 2019 (www.coreldraw.com) was used for the generation of figures.

### Reporting summary

Further information on research design is available in the Nature Research Reporting Summary linked to this article.

## Supplementary information

Supplementary Information

Description of Additional Supplementary Files

Supplementary Data 1

Supplementary Data 2

Reporting summary

## Data Availability

Source data are provided with this paper. All data are also available from the corresponding author on request. Figure1A: LFQ proteome data is provided as Supplementary Data 2 and is deposited under PRIDE PXD030463. Furthermore, the following publicly available datasets were used: GSE13159 (Mile)^54^ and TCGA Cancer Genome Atlas Research^3^ (https://www.cancer.gov/about-nci/organization/ccg/research/structural-genomics/tcga). Source data are provided with this paper.

## Source data

Source data

## References

[1] Miller CA, Wilson RK, Ley TJ (2013). Genomic landscapes and clonality of de novo AML. N. Engl. J. Med..

[2] Welch JS (2012). The origin and evolution of mutations in acute myeloid leukemia. Cell.

[3] Ley TJ (2013). Genomic and epigenomic landscapes of adult de novo acute myeloid leukemia. N. Engl. J. Med..

[4] Corces-Zimmerman MR, Hong WJ, Weissman IL, Medeiros BC, Majeti R (2014). Preleukemic mutations in human acute myeloid leukemia affect epigenetic regulators and persist in remission. Proc. Natl Acad. Sci. USA.

[5] Jan M, Majeti R (2013). Clonal evolution of acute leukemia genomes. Oncogene.

[6] Shlush LI (2014). Identification of pre-leukaemic haematopoietic stem cells in acute leukaemia. Nature.

[7] de Boer B (2018). Prospective isolation and characterization of genetically and functionally distinct AML subclones. Cancer Cell.

[8] Anderson K (2011). Genetic variegation of clonal architecture and propagating cells in leukaemia. Nature.

[9] Klco JM (2014). Functional heterogeneity of genetically defined subclones in acute myeloid leukemia. Cancer Cell.

[10] Hughes AE (2014). Clonal architecture of secondary acute myeloid leukemia defined by single-cell sequencing. PLoS Genet..

[11] Warburg O (1956). On the origin of cancer cells. Science.

[12] Filippi MD, Ghaffari S (2019). Mitochondria in the maintenance of hematopoietic stem cells: new perspectives and opportunities. Blood.

[13] Hao X (2019). Metabolic imaging reveals a unique preference of symmetric cell division and homing of leukemia-initiating cells in an endosteal niche. Cell Metab..

[14] Simsek T (2010). The distinct metabolic profile of hematopoietic stem cells reflects their location in a hypoxic niche. Cell Stem Cell.

[15] Vannini N (2016). Specification of haematopoietic stem cell fate via modulation of mitochondrial activity. Nat. Commun..

[16] Vannini N (2019). The NAD-booster nicotinamide riboside potently stimulates hematopoiesis through increased mitochondrial clearance. Cell Stem Cell.

[17] Yu WM (2013). Metabolic regulation by the mitochondrial phosphatase PTPMT1 is required for hematopoietic stem cell differentiation. Cell Stem Cell.

[18] Ito K (2016). Self-renewal of a purified Tie2+ hematopoietic stem cell population relies on mitochondrial clearance. Science.

[19] de Almeida MJ, Luchsinger LL, Corrigan DJ, Williams LJ, Snoeck HW (2017). Dye-independent methods reveal elevated mitochondrial mass in hematopoietic stem cells. Cell Stem Cell.

[20] Bonora M, Ito K, Morganti C, Pinton P, Ito K (2018). Membrane-potential compensation reveals mitochondrial volume expansion during HSC commitment. Exp. Hematol..

[21] Ito K (2006). Reactive oxygen species act through p38 MAPK to limit the lifespan of hematopoietic stem cells. Nat. Med..

[22] Lagadinou ED (2013). BCL-2 inhibition targets oxidative phosphorylation and selectively eradicates quiescent human leukemia stem cells. Cell Stem Cell.

[23] Khan N (2016). Normal hematopoietic progenitor subsets have distinct reactive oxygen species, BCL2 and cell-cycle profiles that are decoupled from maturation in acute myeloid leukemia. PLoS ONE.

[24] Jones CL (2019). Cysteine depletion targets leukemia stem cells through inhibition of electron transport complex II. Blood.

[25] Adane B (2019). The hematopoietic oxidase NOX2 regulates self-renewal of leukemic stem cells. Cell Rep..

[26] Jones CL (2018). Inhibition of amino acid metabolism selectively targets human leukemia stem cells. Cancer Cell.

[27] Seneviratne AK (2019). The mitochondrial transacylase, tafazzin, regulates for AML stemness by modulating intracellular levels of phospholipids. Cell Stem Cell.

[28] Anso E (2017). The mitochondrial respiratory chain is essential for haematopoietic stem cell function. Nat. Cell Biol..

[29] Takubo K (2013). Regulation of glycolysis by Pdk functions as a metabolic checkpoint for cell cycle quiescence in hematopoietic stem cells. Cell Stem Cell.

[30] Klimmeck D (2012). Proteomic cornerstones of hematopoietic stem cell differentiation: distinct signatures of multipotent progenitors and myeloid committed cells. Mol. Cell Proteom..

[31] Halvarsson C, Eliasson P, Jönsson J-I (2017). Pyruvate dehydrogenase kinase 1 is essential for transplantable mouse bone marrow hematopoietic stem cell and progenitor function. PLoS ONE.

[32] Chen WL (2014). A distinct glucose metabolism signature of acute myeloid leukemia with prognostic value. Blood.

[33] Gregory MA (2017). Glutaminase inhibition improves FLT3 inhibitor therapy for acute myeloid leukemia. Exp. Hematol..

[34] de Jonge HJ (2011). Gene expression profiling in the leukemic stem cell-enriched CD34(+) fraction identifies target genes that predict prognosis in normal karyotype AML. Leukemia.

[35] Bagger FO (2016). BloodSpot: a database of gene expression profiles and transcriptional programs for healthy and malignant haematopoiesis. Nucleic Acids Res.

[36] Rardin MJ, Wiley SE, Naviaux RK, Murphy AN, Dixon JE (2009). Monitoring phosphorylation of the pyruvate dehydrogenase complex. Anal. Biochem.

[37] Korotchkina LG, Patel MS (2001). Site specificity of four pyruvate dehydrogenase kinase isoenzymes toward the three phosphorylation sites of human pyruvate dehydrogenase. J. Biol. Chem..

[38] Qin L (2015). Targeting PDK1 with dichloroacetophenone to inhibit acute myeloid leukemia (AML) cell growth. Oncotarget.

[39] Antonelli A (2016). Establishing human leukemia xenograft mouse models by implanting human bone marrow-like scaffold-based niches. Blood.

[40] Carretta M (2017). Genetically engineered mesenchymal stromal cells produce IL-3 and TPO to further improve human scaffold-based xenograft models. Exp. Hematol..

[41] Huang Q (2018). Characterization of the interactions of potent allosteric inhibitors with glutaminase C, a key enzyme in cancer cell glutamine metabolism. J. Biol. Chem..

[42] Qin L (2016). Targeting PDK1 with dichloroacetophenone to inhibit acute myeloid leukemia (AML) cell growth. Oncotarget.

[43] Jayavelu AK, Moloney JN, Bohmer FD, Cotter TG (2016). NOX-driven ROS formation in cell transformation of FLT3-ITD-positive AML. Exp. Hematol..

[44] Stanicka J, Russell EG, Woolley JF, Cotter TG (2015). NADPH oxidase-generated hydrogen peroxide induces DNA damage in mutant FLT3-expressing leukemia cells. J. Biol. Chem..

[45] Sallmyr A (2008). Internal tandem duplication of FLT3 (FLT3/ITD) induces increased ROS production, DNA damage, and misrepair: implications for poor prognosis in AML. Blood.

[46] Wierenga ATJ (2019). HIF1/2-exerted control over glycolytic gene expression is not functionally relevant for glycolysis in human leukemic stem/progenitor cells. Cancer Metab..

[47] Gallipoli P (2018). Glutaminolysis is a metabolic dependency in FLT3(ITD) acute myeloid leukemia unmasked by FLT3 tyrosine kinase inhibition. Blood.

[48] Gregory MA (2018). Glutaminase inhibition improves FLT3 inhibitor therapy for acute myeloid leukemia. Exp. Hematol..

[49] Baccelli I (2019). Mubritinib targets the electron transport chain complex I and reveals the landscape of OXPHOS dependency in acute myeloid leukemia. Cancer Cell.

[50] Xie SZ (2019). Sphingolipid modulation activates proteostasis programs to govern human hematopoietic stem cell self-renewal. Cell Stem Cell.

[51] Groen RW (2012). Reconstructing the human hematopoietic niche in immunodeficient mice: opportunities for studying primary multiple myeloma. Blood.

[52] Sontakke P (2016). Modeling BCR-ABL and MLL-AF9 leukemia in a human bone marrow-like scaffold-based xenograft model. Leukemia.

[53] Fellmann C (2013). An optimized microRNA backbone for effective single-copy RNAi. Cell Rep..

[54] Kohlmann A (2008). An international standardization programme towards the application of gene expression profiling in routine leukaemia diagnostics: the Microarray Innovations in LEukemia study prephase. Br. J. Haematol..

[55] Gene Ontology Consortium. (2021). The Gene Ontology resource: enriching a GOld mine. Nucleic Acids Res.

[56] Ashburner M (2000). Gene ontology: tool for the unification of biology. The Gene Ontology Consortium. Nat. Genet..

[57] Subramanian A (2005). Gene set enrichment analysis: a knowledge-based approach for interpreting genome-wide expression profiles. Proc. Natl Acad. Sci. USA.

