# Supplementary material for: The Glycolytic Gatekeeper PDK1 defines different metabolic states between genetically distinct subtypes of human acute myeloid leukemia
Source: Nat Commun. 2022 Mar 1;13:1105. doi: 10.1038/s41467-022-28737-3 (PMC8888573; doi:10.1038/s41467-022-28737-3)
Supplement: Supplementary file 1 — Supplementary Information [file 41467_2022_28737_MOESM1_ESM.pdf]

Supplementary material

The Glycolytic Gatekeeper PDK1 defines different metabolic states between genetically distinct subtypes of human acute myeloid leukemia

Ayşegül Erdem[1,2], Silvia Marin[2,3,4], Diego A. Pereira-Martins[1,5] , Roldán Cortés[2], Alan Cunningham[1], Maurien G. Pruis[1], Bauke de Boer[1], Fiona A.J. van den Heuvel[1], Marjan Geugien[1], Albertus T.J. Wierenga[1,6], Annet Z. Brouwers-Vos[1], Eduardo M. Rego[5], Gerwin Huls[1], Marta Cascante[2,3,4] and Jan Jacob Schuringa[1,7].

[1]Department of Experimental Hematology, University Medical Center Groningen, University of Groningen, Hanzeplein 1, 9700 RB, Groningen, The Netherlands.

[2]Department of Biochemistry and Molecular Biology, Faculty of Biology, Avda. Diagonal 643, Barcelona 08028, Spain.

[3]CIBER of Hepatic and Digestive Diseases (CIBEREHD), Institute of Health Carlos III, 28029 Madrid, Spain.

[4]Institute of Biomedicine of University of Barcelona, 08028 Barcelona, Spain

[5]Hematology Division, LIM31, Faculdade de Medicina, University of São Paulo, São Paulo, SP, Brazil.

[6]Department of Laboratory Medicine, University Medical Center Groningen, University of Groningen, Hanzeplein 1, 9700 RB, Groningen, The Netherlands.

[7]Corresponding author

Details corresponding author: Jan Jacob Schuringa, Department of Experimental Hematology, Cancer Research Center Groningen (CRCG), University Medical Center Groningen, University of Groningen, Hanzeplein 1, DA13, 9700RB, Groningen, The Netherlands. Phone: +31-503619391, fax: +31-503614862, email: j.j.schuringa@umcg.nl

SUPPLEMENTARY FIGURES

Supplementary Figure 1

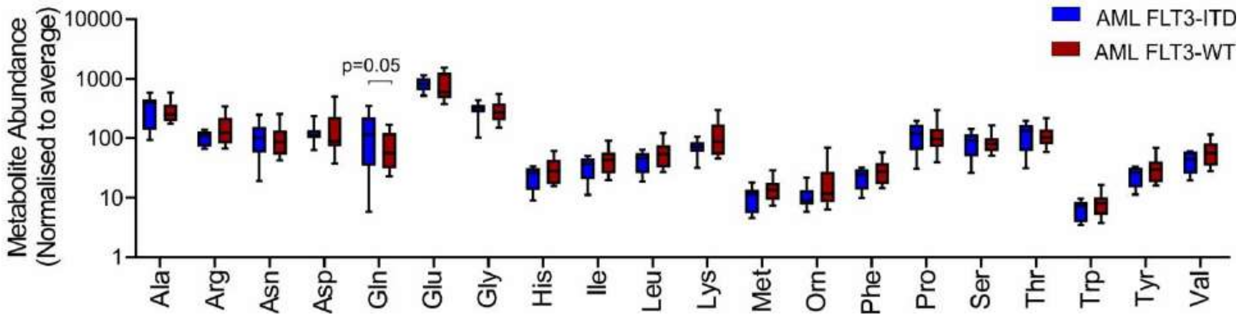

Supplementary Fig. 1. Comparison of amino acid abundance in FLT3-ITD and wt AML subtypes. A box and whisker plot (min to max) showing relative internal distribution of each amino acid abundance (pmol/micrograms) in FLT3-ITD mutated AML primary CD34$^+$ cells (n=10) compared to FLT3-ITD wild type AML primary CD34$^+$ cells (n=15) (log10). The line represents median values. p value is derived from student's t test (two-sided).

Supplementary Figure 2

Acylcarnitines

a

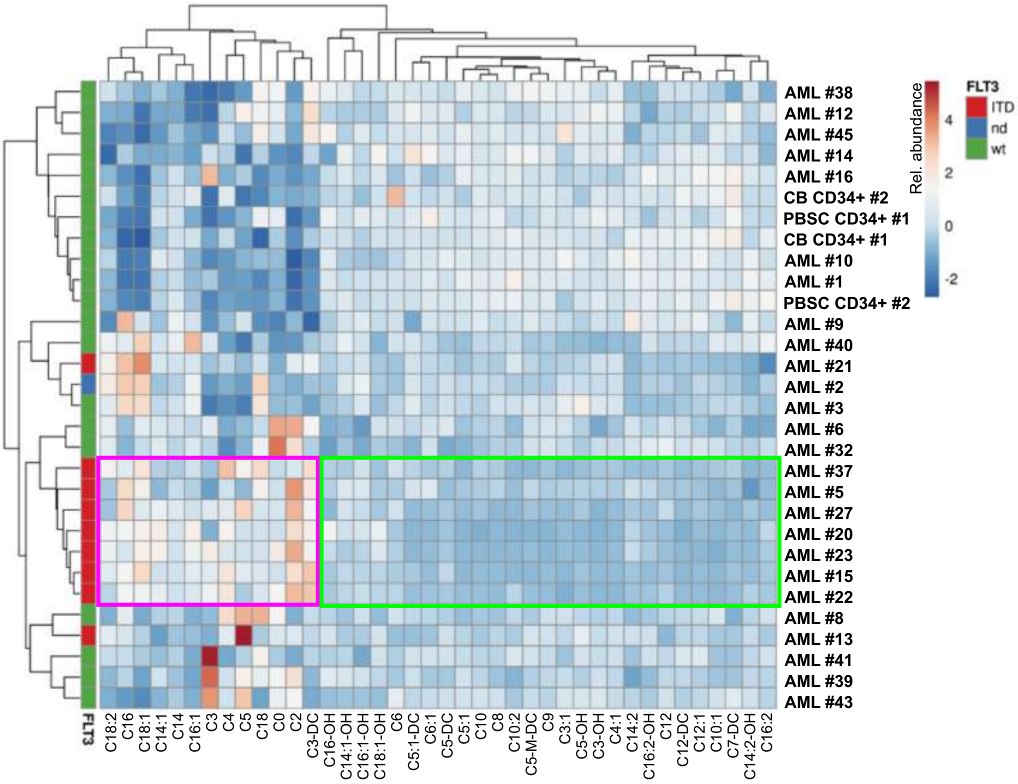

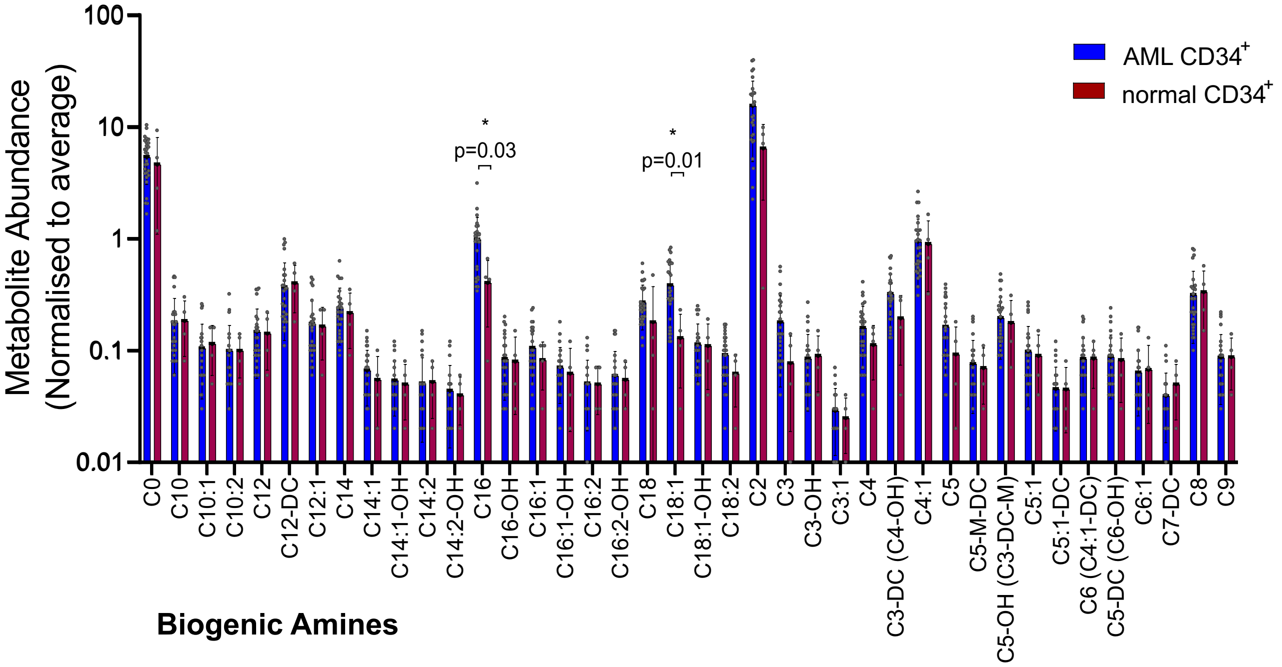

b

Biogenic Amines

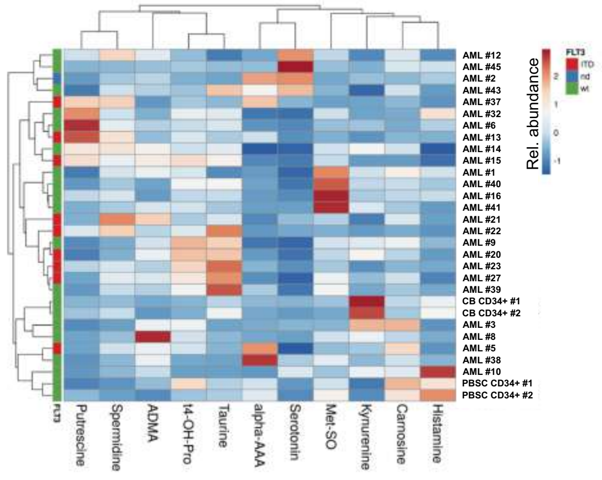

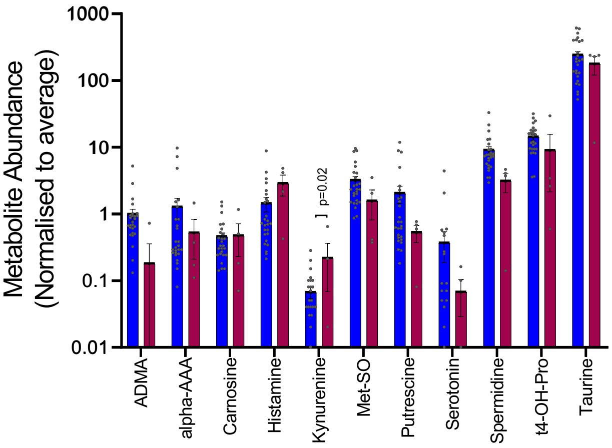

Supplementary Figure 2

c **Sphingolipids**

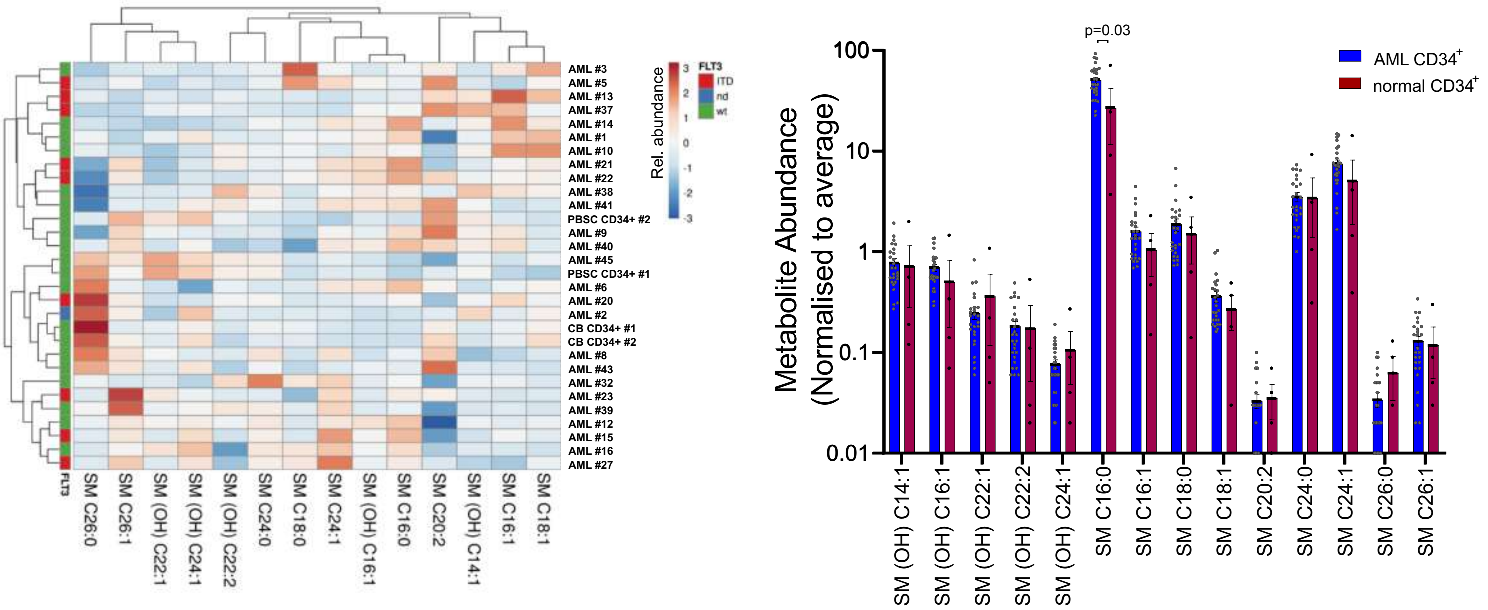

d **Glycerophospholipids**

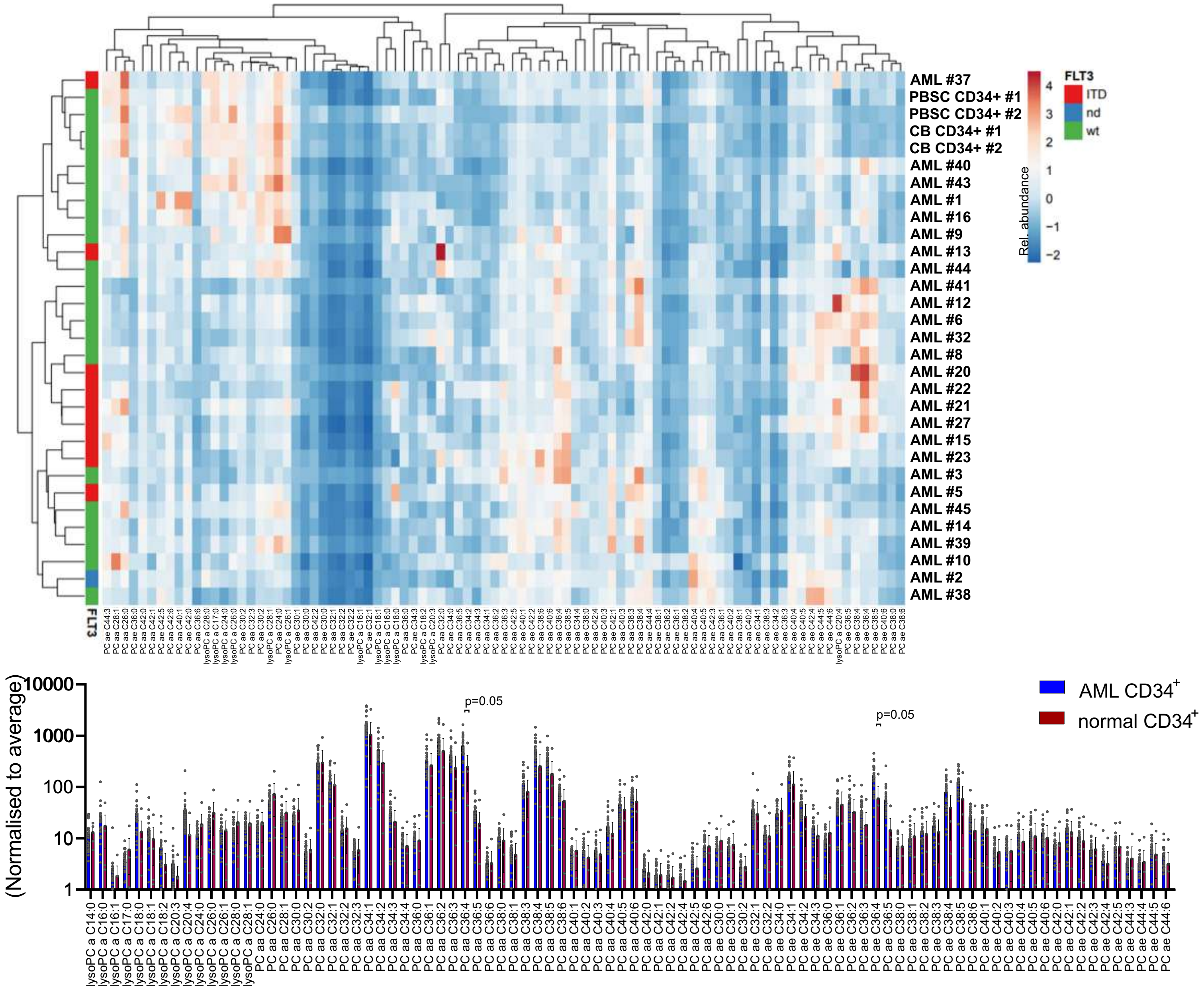

Supplementary Fig. 2. Detailed LC-MS/MS targeted metabolome screen comparison. Heat map of acylcarnitines family (**a**), biogenic amines (**b**), Sphingolipids (**c**), Glycerophospholipids (includes phosphatidylcholines (PC) and lysophosphatidylcholines (lysoPC), a refers to acyl bonds, aa refers to diacyl bonds and ae refers to acyl/ether bonds) (**d**) (x-axis) and their associations with $CD34^+$ sorted AML primary individuals (n=27) and healthy PB (Peripheral blood mobilized stem cells) and CB (Cord Blood) samples (n=4). y-axis indicates genetic backgrounds of AML patients with either FLT3-ITD mutated or wild type. Color code for higher abundance is red and for lower abundance is blue (each value was normalized to the average of each metabolite in each AML or healthy group). Colored rectangles highlight major heterogeneous differences in the corresponding metabolism family. Bar graphs shows detailed comparison of acylcarnitine (a), biogenic amines (b), Sphingolipids (c), Glycerophospholipids (d) internal abundance (log 10) in primary AML versus total healthy PB (Peripheral blood mobilized stem cells) and CB (Cord Blood) $CD34^+$ cells. The bar graphs represent mean+/- SEM and each dot shows individual primary sample. Statistical analysis was performed using multiple t test (two-sided). * $p<0.05$; ** $p<0.01$.

Supplementary Figure 3

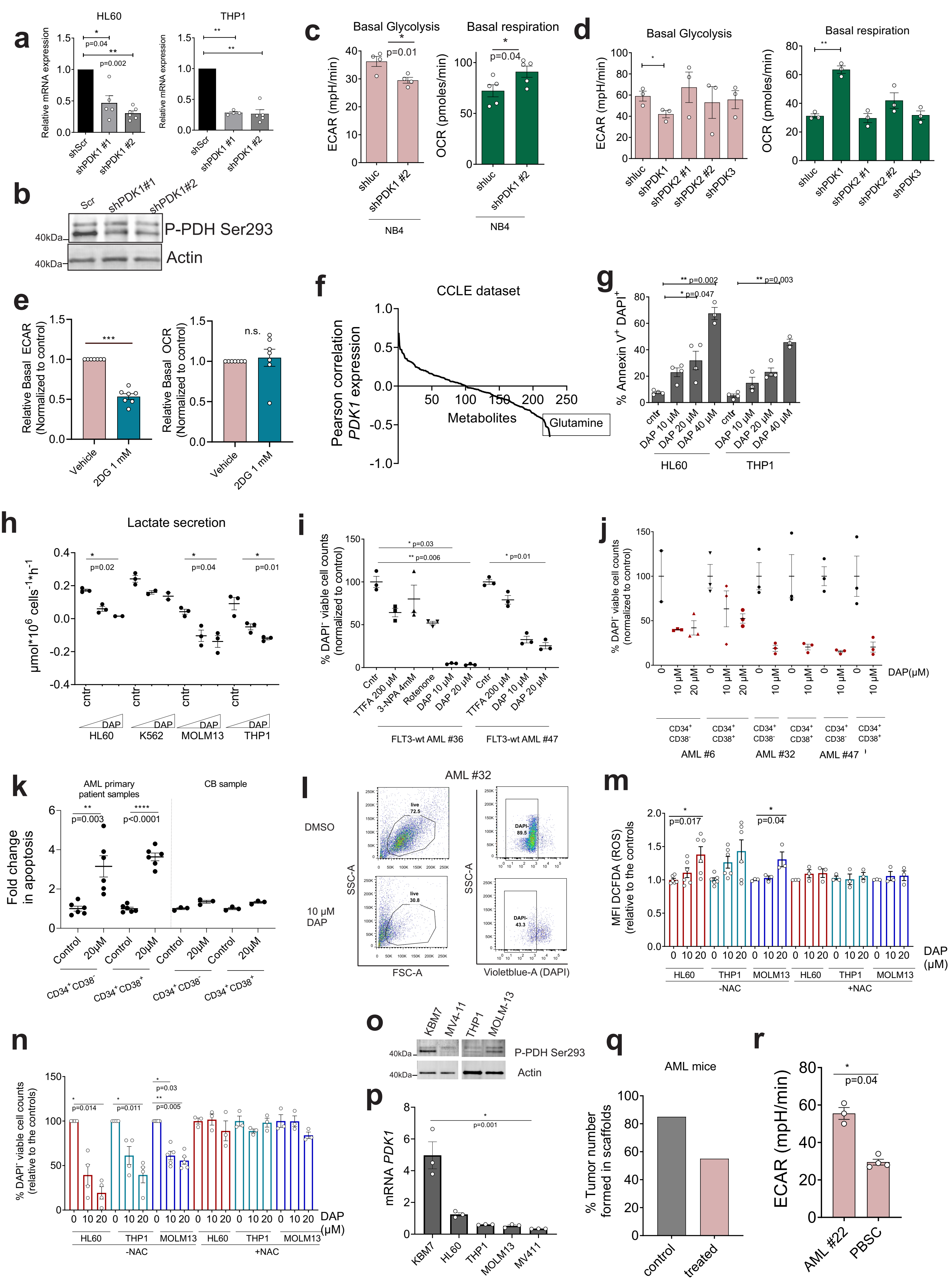

Supplementary Fig. 3. a, *PDK1* mRNA levels in shScr, shPDK1#1, and shPDK1#2 (each dot= biological replicates). **b,** Western blot analysis in HL60 cells. (Representative of two independent replicates)**. c,** Basal ECAR and OCR in GFP^+ sorted shLuc, shPDK1#1, shPDK1#2 NB4 cells (n=4, 5 independently, in quadruplicates) and **d,** in GFP^+-sorted shLuc, shPDK1#1, shPDK2#1, shPDK2#2, shPDK3 HL60 cells (n=3 independently, in quadruplicates) (p=0.04, p=0.004). **e,** as in d, HL60 cells were treated with/without 2DG (24 h). (n=2 independently, measured in quadruplicates) (p=0.0006). **f,** Pearson correlation of PDK1 expression in AML cells compared to metabolites abundance (CCLE dataset). **g,** Apoptosis after 10, 20,40 μM DAP (24 hours) in HL60, THP1 cells. (mean +/- SEM). **h,** Lactate secretion per $1x10^6$ cells/hour in AML cells after 10, 20 μM (24 hours) (Each dot=independent replicates, in triplicates). **i,** (%) $DAPI^-$ cells in AML primary FLT3-wt cells (n=2, technical triplicates) upon 10, 20 μM DAP or 200 mM TTFA or 4 mM 3-Nitropropionicacid or 20 μM Rotenone (2 days). **j,** Relative $DAPI^-$ cells upon 10, 20 μM DAP (3 days) in AML primary $CD34^+$ $CD38^-$ and $CD34^+$ $CD38^+$ (n=3 AMLs, in technical triplicates) cells on MS5-stroma. **k**, Fold change in % Annexin V^+-$DAPI^+$ in $CD34^+/CD38^-$ and $CD34^+/CD38^+$ fractions upon 20 μM DAP (3 days) in AML primary cells (two biological replicates, in technical triplicates) or CB (technical triplicates). **l,** Representative flow-cytometry gating strategy for live and DAPI- cells in primary AML cells and cell lines. **m,** Mean fluorescent DCFH-DA (ROS) intensity and **n**, (%) $DAPI^-$ after 10, 20 μM DAP (24 hours) in AML cells (n=3-6 independently, in technical triplicates) in the absence/presence of 2 mM N-acetyl-l-cysteine. **o**, Westernblot analysis of P-PDH-Ser293 and Actin in AML cells. (Representative of independent triplicates). **p**, *PDK1* mRNA levels in AML cells. (n=3 independently). **q**, (%) total tumor numbers formed in the scaffolds compared to initial injections, before and

after the treatment in AML primary-xenograft mice. **r**, ECAR in CD34$^+$-sorted AML#22 (n=1, in quadruplicates) and PBMSCs (n=1, in quadruplicates). Error bars=mean +/- SEM. (c, e, k, q) Student's t-test (two-sided) or (a, d, g, h, i, j, l, m, o) Kruskal-Wallis one-way ANOVA test.

Supplementary Figure 4

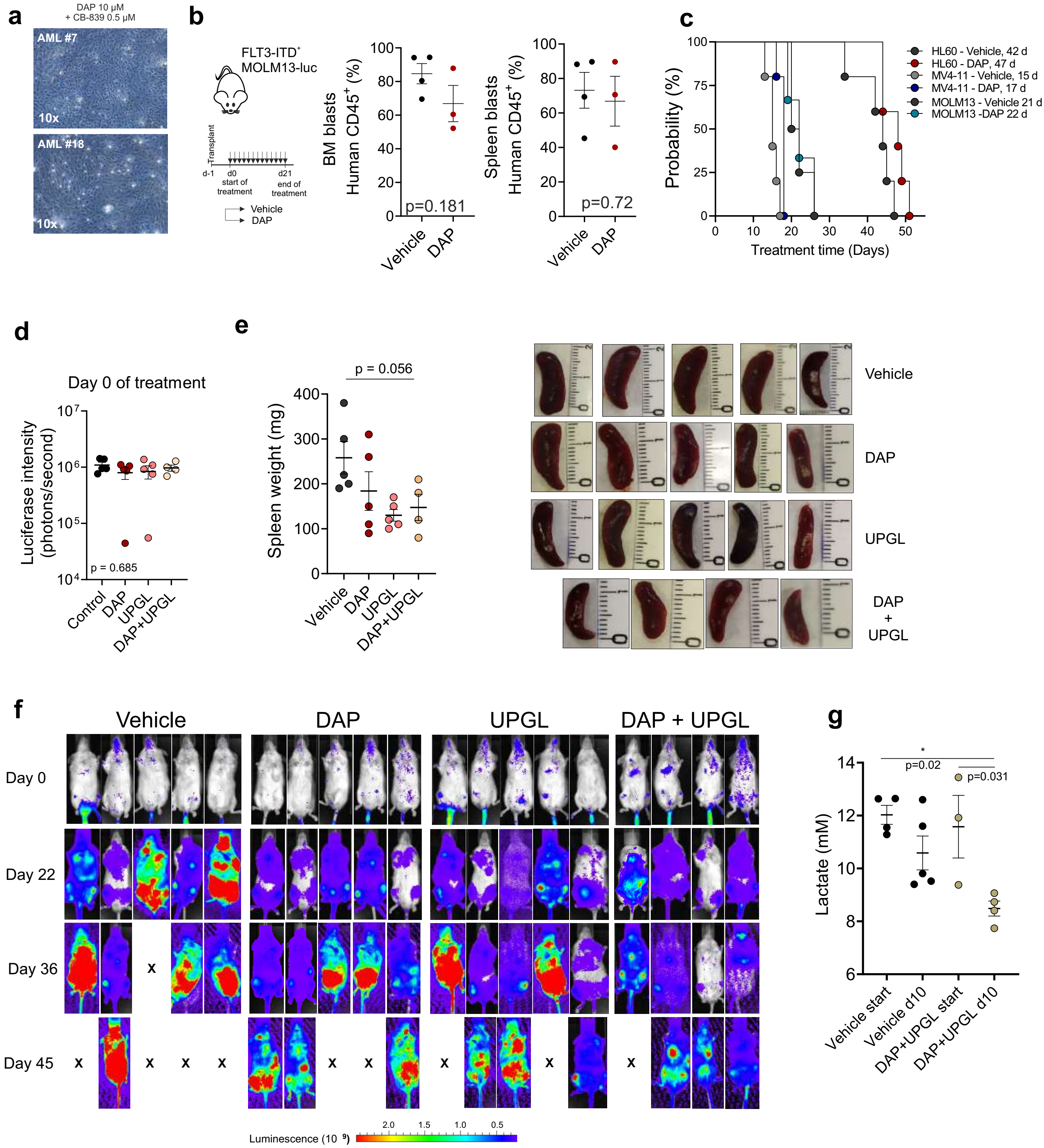

Supplementary Fig. 4. a, Representative microscopic image of remaining MS5 murine stromal cells after $CD34^+$ AML primary cell culture were washed out (n=2 AMLs) after co-treatment of DAP (10 μM) and CB-839 (0.5 μM) at 10x magnification. **b**, FLT3-ITD^+ MOLM13 cells were i.v. injected in NSG mice and mice were daily treated intraperitoneally with vehicle and DAP (20 mg/kg) (n=4 or 3 in each group). Human $CD45^+$ % in BM (left panel) and spleen (right panel) at the time of sacrifice is shown. Data expressed as mean +/- standard error of the mean (SEM). **c**, Kaplan–Meier curve of mice transplanted with MV411, MOLM13 and HL60 cells treated with DAP (20 mg/kg) or vehicle. All mice showed clear evidence of leukemia in blood, BM and spleen at moment of sacrifice. P value (by Log-rank test) indicate statistical significance for comparisons of DAP treated mice with control mice (vehicle) (p=0.03 for MV411, p=0.92 for MOLM13 and p= 0.05 for HL60). **d**, Luciferase intensity in mice transplanted with HL60 cells at the start of the treatment at the start of the treatment (vehicle or DAP (20 mg/kg, i.p.) or UPGL00004 (indicated as UPGL; 3mg/kg, i.p.) or with combination of DAP and UPGL00004 after 7 days of transplantation (n=5 or 4, in each group) . Each data point represents mean +/- SEM. **e**, Spleen weights at the time of sacrifice in HL60 mice, data expressed as mean +/- standard error of the mean (SEM) (left panel) and right panel shows photographic images of corresponding spleen sizes of HL60 mice treatment and vehicle groups. **f**, On days 0, 22, 36 and 45 luciferase signals were detected by IVIS imaging in different treatment groups of mice transplanted with luciferase expressing HL60 cells. **g**, Spectrophotometric lactate measurement on plasma samples from mice transplanted with HL60 and treated with combination of DAP and UPGL and with PBS(vehicle) on day 0 and day 10 (d10). Dots represent individual mouse samples as mean values of assessed in three biological replicates. Error bars

represent mean +/- SEM. (b) Student's t test (two-sided) or (d, e, g) Kruskal-Wallis one-way ANOVA test for multiple comparisons.

Supplementary Figure 5. Full western blot scans

Figure 3f

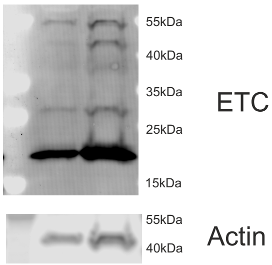

Figure 4d

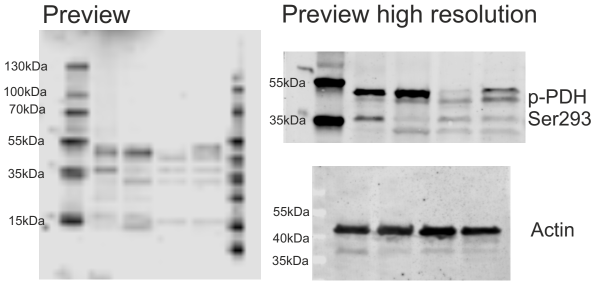

Figure 5f

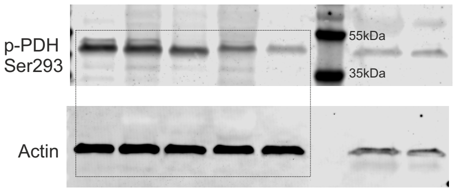

Figure 5a

THP1 and MOLM13

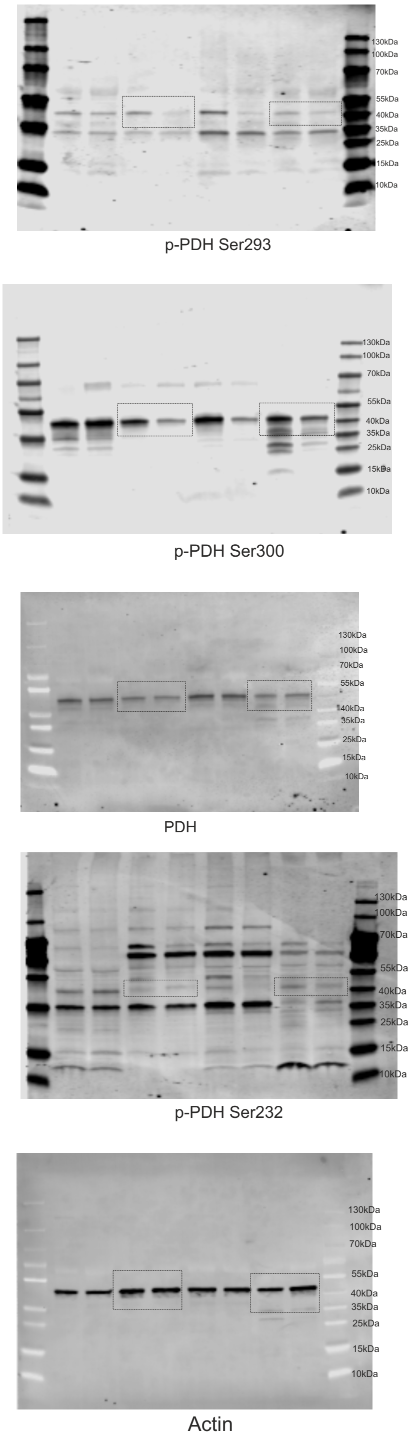

Figure 5a

HL-60

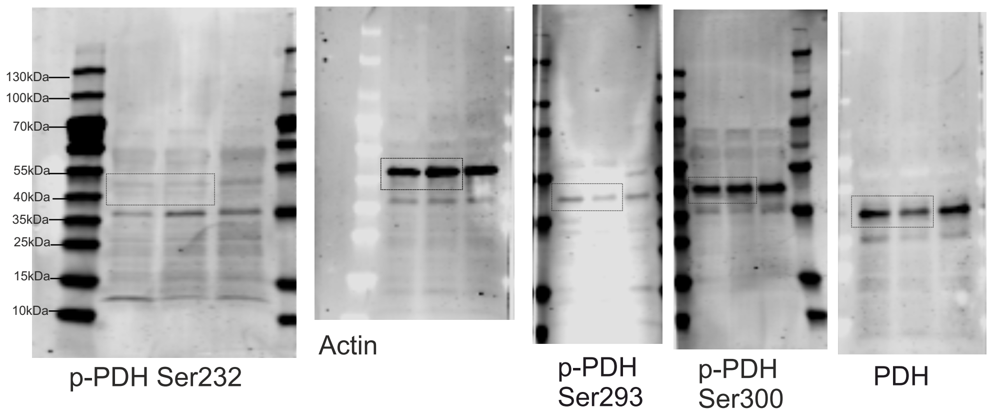

Supplementary Figure 3b

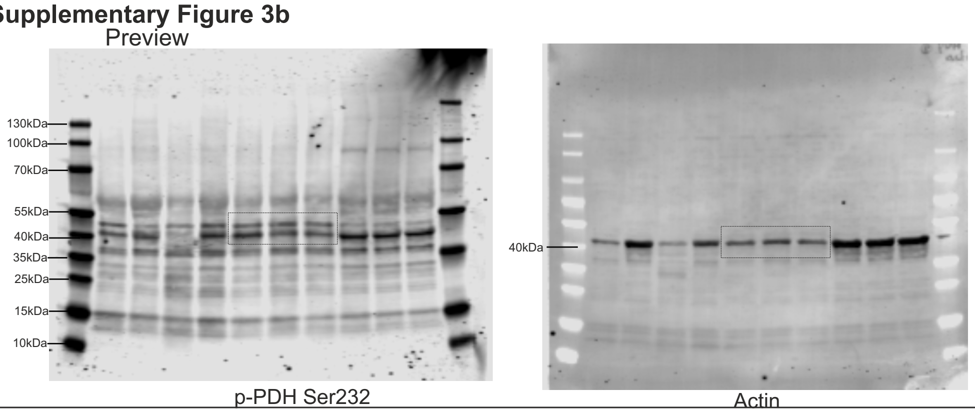

Supplementary Figure 3n

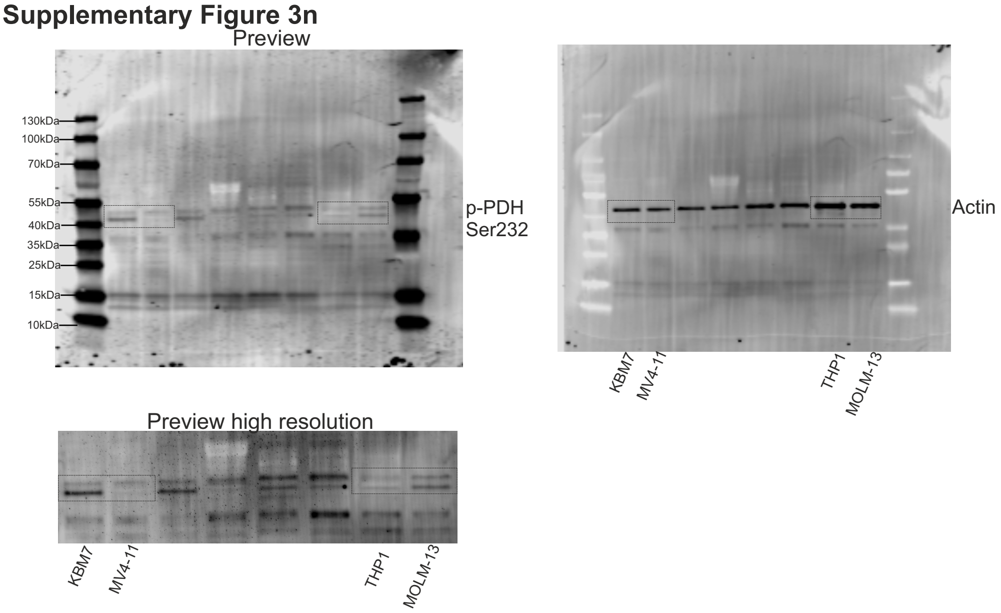

Supplementary Fig. 5. Western blot of raw scans from main and extended data files**.** Each corresponding data panel is indicated inside a black rectangle. Images in Figure 4d, 5f, Extended data Figure 3b and Extended Figure 3n are derived from the same blot. MOLM13 and THP1 cell lines protein expressions were ran and analyzed in the same blot and a separate blot was used for HL60 cell line analysis. PDH staining in Figure 5a was stripped and reblotted from the same blot in both HL60 blot and THP1and MOLM13 blots.
