# Supplementary material for: The Glycolytic Gatekeeper PDK1 defines different metabolic states between genetically distinct subtypes of human acute myeloid leukemia
Source: Nat Commun. 2022 Mar 1;13:1105. doi: 10.1038/s41467-022-28737-3 (PMC8888573; doi:10.1038/s41467-022-28737-3)
Supplement: Supplementary file 2 — Description of Additional Supplementary Files [file 41467_2022_28737_MOESM2_ESM.pdf]

Description of Additional Supplementary Files

Supplementary Data 1: Patient characteristics

Supplementary Data 2: LFQ Proteome data (log 10)
