# Supplementary material for: The Glycolytic Gatekeeper PDK1 defines different metabolic states between genetically distinct subtypes of human acute myeloid leukemia
Source: Nat Commun. 2022 Mar 1;13:1105. doi: 10.1038/s41467-022-28737-3 (PMC8888573; doi:10.1038/s41467-022-28737-3)
Supplement: Supplementary file 5 — Reporting summary [file 41467_2022_28737_MOESM5_ESM.pdf]

nature research

Corresponding author(s): Jan Jacob Schuringa

Last updated by author(s): January 24th 2022

Reporting Summary

Nature Research wishes to improve the reproducibility of the work that we publish. This form provides structure for consistency and transparency in reporting. For further information on Nature Research policies, seeAuthors & Referees and theEditorial Policy Checklist .

Statistics

For all statistical analyses, confirm that the following items are present in the figure legend, table legend, main text, or Methods section.

n/a	Confirmed	
☐	☒	The exact sample size (*n*) for each experimental group/condition, given as a discrete number and unit of measurement
☐	☒	A statement on whether measurements were taken from distinct samples or whether the same sample was measured repeatedly
☐	☒	The statistical test(s) used AND whether they are one- or two-sided<br>*Only common tests should be described solely by name; describe more complex techniques in the Methods section.*
☒	☐	A description of all covariates tested
☒	☐	A description of any assumptions or corrections, such as tests of normality and adjustment for multiple comparisons
☐	☒	A full description of the statistical parameters including central tendency (e.g. means) or other basic estimates (e.g. regression coefficient) AND variation (e.g. standard deviation) or associated estimates of uncertainty (e.g. confidence intervals)
☐	☒	For null hypothesis testing, the test statistic (e.g. *F*, *t*, *r*) with confidence intervals, effect sizes, degrees of freedom and *P* value noted<br>*Give P values as exact values whenever suitable.*
☒	☐	For Bayesian analysis, information on the choice of priors and Markov chain Monte Carlo settings
☐	☒	For hierarchical and complex designs, identification of the appropriate level for tests and full reporting of outcomes
☐	☒	Estimates of effect sizes (e.g. Cohen's *d*, Pearson's *r*), indicating how they were calculated

Our web collection on statistics for biologists contains articles on many of the points above.

Software and code

Policy information about availability of computer code

Data collection	no software was used for data collection
Data analysis	The following software was used for data analyses:<br>Corel draw 2019: Corel TM (www.coreldraw.com) was used for the generation of Figures.<br>Prism 8 Graphpad (www.graphpad.com) was used for statistical tests for all bar graphs<br>FlowJo v10.0.6 TreeStar (www.flowjo.com) was used for ana;yse flow cytometry data<br>LC-MS/MS data was analyzed using Analyst 1.7 (AbSciex, Framingham, MA, USA)<br>MetIDQ™ Biocrates Life Sciences (www.biocrates.com) was used for analyzing Mass Spectrometry Based Targeted Metabolomics Assay generated data.<br>MyIQ Bio-Rad (www.bio-rad.com) was used for analyzing Q-RT-PCR data<br>ClustVis (BETA) (https://biit.cs.ut.ee/clustvis/) was used to generate clustering heatmaps.<br>statistical package for the social sciences (SPSS) 19.0 was used for statistical tests<br>For GSEA studies, the GenePattern GSEAPreranked v4.1.0 module was used and statistical results are included as part of the default paramaters.

For manuscripts utilizing custom algorithms or software that are central to the research but not yet described in published literature, software must be made available to editors/reviewers. We strongly encourage code deposition in a community repository (e.g. GitHub). See the Nature Research guidelines for submitting code & software for further information.

Data

Policy information about availability of data

All manuscripts must include a data availability statement. This statement should provide the following information, where applicable:

- Accession codes, unique identifiers, or web links for publicly available datasets
- A list of figures that have associated raw data
- A description of any restrictions on data availability

Source data are provided with this paper. All data are also available from the corresponding author on request. Fig.1A: LFQ proteome data is provided as Supplemental Table 2 and is available under PRIDE PXD030463. Furthermore, the following publicly available datasets were used: GSE13159 (Mile) and TCGA Cancer Genome Atlas Research3 (https://www.cancer.gov/about-nci/organization/ccg/research/structural-genomics/tcga)

Field-specific reporting

Please select the one below that is the best fit for your research. If you are not sure, read the appropriate sections before making your selection.

- [x] Life sciences
- [ ] Behavioural & social sciences
- [ ] Ecological, evolutionary & environmental sciences

For a reference copy of the document with all sections, see nature.com/documents/nr-reporting-summary-flat.pdf

Life sciences study design

All studies must disclose on these points even when the disclosure is negative.

Sample size	Cell biological experiments were performed in triplicate (or more when indicated in the Figures). No statistical method was used to pre-determine sample size, but our sample size was similar to previous studies since in our experience this is sufficient to determine reproducible results. For the quantitative proteome studies, 6 healthy PB CD34+ samples and 42 AML patient samples were included. Also here, no statistical method was used to pre-determine sample size.
Data exclusions	No data were excluded from analyses
Replication	All data are from a minimum of 3 independent experiments
Randomization	Samples were randomized during data collection. For MOLM13, MV4-11 and HL60 in vivo engraftment studies, animals were randomly assigned to treatment or not treatment groups based on IVIS results ensuring that the average engraftment levels in each group were comparable. For the HuScaffold in vivo KBM7 and PDX models , mice were randamly assigned to treatment or non-treatment groups.
Blinding	Investigators were not blinded to the experiments as the researchers need to collect samples based on the treatment and cell type information.

Reporting for specific materials, systems and methods

We require information from authors about some types of materials, experimental systems and methods used in many studies. Here, indicate whether each material, system or method listed is relevant to your study. If you are not sure if a list item applies to your research, read the appropriate section before selecting a response.

Materials & experimental systems

n/a	Involved in the study	
[ ]	[x]	Antibodies
[ ]	[x]	Eukaryotic cell lines
[x]	[ ]	Palaeontology
[ ]	[x]	Animals and other organisms
[ ]	[x]	Human research participants
[x]	[ ]	Clinical data

Methods

n/a	Involved in the study	
[x]	[ ]	ChIP-seq
[ ]	[x]	Flow cytometry
[x]	[ ]	MRI-based neuroimaging

Antibodies

Antibodies used	CD34-APC BD Biosciences Cat#555824 (3 µL/100 µL)<br>CD38-PE BD Biosciences Cat#345806 (3 µL/100 µL)<br>Annexin V-FITC Miltenyi Biotech Cat#130-093-060 (0.5 µL/100 µL)<br>CD45-PerCP Biolegend Cat#3040256 (2 µL/100 µL)<br>CD33 APC Biolegend Cat#303408 (5 µL/100 µL)

	CD11b APC Biolegend Cat#101211 (2 µL/100 µL)<br>CD14 PE Biolegend Cat#325606 (2 µL/100 µL)<br>CD15 APC BD Biosciences Cat#551376 (2 µL/100 µL)<br>CD13 PE BD Biosciences Cat#555394 (2 µL/100 µL)<br>CD3 APC BD Biosciences Cat#555335 (5 µL/100 µL)<br>CD117 PE BD Biosciences Cat#332785 (3 µL/100 µL)<br>CD19 PE BD Biosciences Cat#345777 (5 µL/100 µL)<br>CD34 FITC BD Biosciences Cat#555821 (3 µL/100 µL)<br>P-PDH Ser 232 (rabbit, Millipore AP1063) (1:1500)<br>P-PDH Ser 300 (rabbit, Millipore, Calbiochem AP1064) (1:1500)<br>P-PDH Ser 293 (Millipore, Calbiochem AP1062) (1:1500)<br>PDH E1 alpha (mouse, Abcam, ab110330) (1:1000)<br>Actin (mouse, Santa Cruz Biotechnology, SC-47778) (1:3000)<br>Actin (rabbit, Cell Signaling Technology, 4970) (1:3000)<br>Alexa Fluor 6 goat anti rabbit (Invitrogen, A21109) (1:3000)<br>IRDye 800CW donkey anti mouse (Li-Cor 926-32212) (1:3000)
Validation	All these antibodies have been validated (information can be found on the vendor's websites). CD34-APC BD Biosciences Cat#555824; CD38-PE BD Biosciences Cat#345806; Annexin V-FITC Miltenyi Biotech Cat#130-093-060 (0.5 µL/100 µL);D45-PerCP Biolegend Cat#3040256; CD33 APC Biolegend Cat#303408 (5 µL/100 µL);CD11b APC Biolegend Cat#101211;CD14 PE Biolegend Cat#325606; CD15 APC BD Biosciences Cat#551376; CD13 PE BD Biosciences Cat#555394 ; CD3 APC BD Biosciences Cat#555335; CD117 PE BD Biosciences Cat#332785 ; CD19 PE BD Biosciences Cat#345777; CD34 FITC BD Biosciences Cat#555821; P-PDH Ser 232 (rabbit, Millipore AP1063); P-PDH Ser 300 (rabbit, Millipore, Calbiochem AP1064); P-PDH Ser 293 (Millipore, Calbiochem AP1062); PDH E1 alpha (mouse, Abcam, ab110330); Actin (mouse, Santa Cruz Biotechnology, SC-47778); Actin (rabbit, Cell Signaling Technology, 4970); Alexa Fluor 6 goat anti rabbit (Invitrogen, A21109); IRDye 800CW donkey anti mouse (Li-Cor 926-32212)

Eukaryotic cell lines

Policy information about cell lines

Cell line source(s)	HL60 ATCC ATCC® CCL-240™<br>THP1 ATCC ATCC® TIB-202™<br>MOLM13 DSMZ CAT#ACC 554<br>K562 ATCC ATCC® CCL-243<br>NB4 DSMZ Cat#ACC 207<br>OCIAML3 DSMZ Cat#ACC 582<br>MV411 ATCC ATCC® CRL-9591<br>KBM7 Gift from the Brummelkamp lab (https://www.nki.nl/divisions/biochemistry/brummelkamp-t-group/)
Authentication	Commercially available cell lines were obtained from the ATCC or DSMZ. Cell lines were not externally authenticated thereafter.
Mycoplasma contamination	Our cell lines routinely tested negative for mycoplasma contamination.
Commonly misidentified lines (See ICLAC register)	No cell lines used in this study were found in the database of commonly misidentified cell lines that is maintained by ICLAC and NCBI Biosample.

Animals and other organisms

Policy information about studies involving animals; ARRIVE guidelines recommended for reporting animal research

Laboratory animals	6- to 8-week-old female NOD.Cg-PrkdcscidIl2rgtm1Wjl/SzJ (NSG) mice were used for the experiments. All animals were housed under specific pathogen free conditions in individually ventilated cages during the whole experiment and were maintained according to the Guide for Care and Use of Laboratory Animals of the National Research Council, USA, and to the National Council of Animal Experiment Control recommendations.
Wild animals	The study did not involve wild animals
Field-collected samples	The study did not involve samples collected from the field
Ethics oversight	All our animal studies were performed in accordance with national and institutional guidelines. All experiments were approved by the Animal Ethics Committee of the University of Sao Paulo and by Central Animal Facility University Medical Center Groningen (#067/2018).

Note that full information on the approval of the study protocol must also be provided in the manuscript.

Human research participants

Policy information about studies involving human research participants

Population characteristics	All patients had acute myeloid leukemia diagnosed in accordence with World health Organization guidelines at the time of diagnosis. Patient characteristics are described in the methods section and Supplementary Table 1. Neonatal Cord blood (CB) samples were obtained from healthy full-term pregnancies.
Recruitment	Samples were stored and obtained via the UMCG biobank
Ethics oversight	Neonatal Cord blood (CB) samples were obtained from healthy full-term pregnancies and AML specimens were obtained from apheresis product, peripheral blood, or bone marrow from AML patients and mobilized peripheral blood from healthy donors who gave informed consent about procedures were obtained in accordance with the Declaration of Helsinki at the obstetrics departments at the Martini Hospital and University Medical Center Groningen. The study was approved by the UMCG Medical Ethical Committee.

Note that full information on the approval of the study protocol must also be provided in the manuscript.

Flow Cytometry

Plots

Confirm that:

☒ The axis labels state the marker and fluorochrome used (e.g. CD4-FITC).

☒ The axis scales are clearly visible. Include numbers along axes only for bottom left plot of group (a 'group' is an analysis of identical markers).

☒ All plots are contour plots with outliers or pseudocolor plots.

☒ A numerical value for number of cells or percentage (with statistics) is provided.

Methodology

Sample preparation	Prior to antibody staining, cells were blocked with anti-human FcR Block (Mylteni Biotech) and murine cells were blocked with anti-Fc (BD Biosciences). For in vivo studies, tumor cells were stained with anti-CD34, anti-CD38, anti-CD45, anti-CD33, anti-CD11b, anti-CD14, anti-CD15, anti CD-13, anti-CD3, anti-CD117 and anti-CD19 antibodies (data not shown) at 4°C for 30 min followed by DAPI- staining for 10 min . Samples washed twice in PBS before each flow cytometric measurements. Fluorescence was measured on the MACSQuant Analyzer 10 (Miltenyi Biotech). Primary AML, Healthy CD34 + CB and PBMSCs cells were stained with anti-CD34 and anti-CD38 antibodies prior to the flow cytometric and sorting experiments.<br>Apoptosis was quantified with Annexin V staining (FITC) according to manufacturer's protocol (Mylteni Biotech). Equal number of cells are stained with Annexin V in calcium supplied sterile water buffer at a concentration of 1 x 106 cells/ml for 20 min at 4°C in dark followed by DAPI- staining for 10 min.<br>For ROS measurements, 2′,7′-dichlorofluorescein diacetate (DCF-DA) (Sigma Aldrich) was dissolved as a 10 mM stock solution in DMSO. Cells were resuspended in PBS containing the probe at a 10μM final concentration. Cells were stained for 30 min. at 37°C in dark. Accumulation of DCF in cells was measured by an increase in fluorescence at 530 nm when the sample is excited at 485 nm (FITC channel). Fluorescence was measured on the MACSQuant Analyzer 10 (Miltenyi Biotech). ROS scavenger N-Acetyl-L-Cysteine (NAC) (Sigma Aldrich, Steinheim, Germany) was freshly prepared prior to each experiment and used in 2mM final concentration in culture medium.
Instrument	Cell sorting for AML primary stained samples and cell lines GFP+ isolation was performed on a MoFlo-Astrios (Beckman Coulter). Flow cytometric analyses, cell counting and viability measurements were determines on either a LSR-II (BD Biosciences), BD Accuri C6 (Beckton Dickinson, Breda, the Netherlands) or MACSQuant (Miltenyi Biotech) flow cytometer.
Software	All flow cytometery data was analyzed using FlowJo v10.0.6 software (TreeStar, Ashland, OR).
Cell population abundance	A minimum of 10,000 events were colected for each replicate. Single cells were defined by cell size using FSC-A and SSC-W. Details are provided in the Methods section.
Gating strategy	For all flow cytometry experiments cells were first identified by FSC/SSC such that all cells were visible on the plot. Positive populations were determined by comparing experimental samples against unstained controls.

☒ Tick this box to confirm that a figure exemplifying the gating strategy is provided in the Supplementary Information.
